# Vaginal microbiome and inflammation cytokines among Chinese women

**DOI:** 10.1038/s41522-026-00994-w

**Published:** 2026-05-20

**Authors:** Yichan Zhang, Ina Schuppe-Koistinen, Huarui Wang, Xiangyang Tian, Wei-Hua Chen, Guolan Gao, Juan Du

**Affiliations:** 1 Department of Microbiology, Tumor and Cell Biology (MTC), Karolinska Institutet, Stockholm, Sweden; 2 Key Laboratory of Molecular Biophysics of the Ministry of Education, Hubei Key Laboratory of Bioinformatics and Molecular Imaging, Center for Artificial Intelligence Biology, Department of Bioinformatics and Systems Biology, College of Life Science and Technology, Huazhong University of Science and Technology, Wuhan, Hubei China; 3 Neurosurgery, The Affiliated People’s Hospital of Xinxiang Medical University, Xinxiang Medical University, Xinxiang, Henan China; 4 Gynecology Department, West China Second University Hospital, Sichuan University, Chengdu, Sichuan China

**Keywords:** Microbiology, Health care

## Abstract

Human papillomavirus (HPV) infection, cervical intraepithelial neoplasia (CIN), pelvic organ prolapse (POP), cervical polyps, abnormal uterine bleeding, and vaginitis are common gynecological disorders. This study characterized the vaginal microbiome and its cytokine-mediated immune interactions in Chinese women with gynecological disorders. Vaginal swabs from 310 patients diagnosed with gynecological disorders and 112 healthy controls were analyzed via 16S rRNA gene sequencing and cytometric bead array (CBA) to evaluate proinflammatory cytokines. Findings on correlation were validated through in vitro co-culture experiments. HPV infection and CIN were associated with dysbiotic microbial profiles and elevated levels of IL-1α, IL-1β, IL-6, IL-8, MCP-1, and MIG. Stratification by microbial composition demonstrated that HPV/CIN patients with non-*Lactobacillus*-dominant microbiomes harbored the highest IL-1α and IL-1β concentrations. Validated through computational modeling and in vitro analyses, *Lactobacillus crispatus* and *Lactobacillus iners* were identified as being strongly inversely correlated with IL-1α and IL-1β expression. Conversely, non-*Lactobacillus* taxa, including *Bifidobacterium breve*, *Prevotella bivia*, *Gardnerella vaginalis, Sneathia amnii, Sneathia sanguinegens, Prevotella amnii, Escherichia coli*, and *Chlamydia trachomatis*, exhibited positive correlations with proinflammatory cytokines. These findings highlight a connection between *Lactobacillus* species and reduced inflammation, while non-*Lactobacillus* dominance is linked to increased cytokine-driven responses. This study provides valuable insights into microbiome-immune crosstalk in gynecological diseases.

## Introduction

There are millions of gynecological disease cases annually worldwide, with Human Papillomavirus (HPV), cervical intraepithelial neoplasia (CIN), pelvic organ prolapse (POP), cervical polyps (CP), abnormal uterine bleeding (AUB), and vaginitis among the most common^1^. These gynecological conditions significantly impact women’s health and quality of life. Cervical cancer (CC) is a malignant tumor originating in the cervix primary caused by HPV infection^2–5^. CIN is a precancerous lesion strongly associated with the development of invasive cervical cancer and includes both cervical dysplasia and carcinoma in situ. POP occurs when the fibromuscular support structures of the pelvic organs weaken or fail, leading to abnormal positioning of the bladder, uterus, rectum, and/or small intestine inside or outside the vagina^6–8^. POP is a common condition in women, with a prevalence ranging from 30% to 50% among adults, and its incidence increases with age and parity^9–11^. CP are benign lesions found in 2–5% of adult women and these growths are considered as a result of focal hyperplasia of the glandular epithelium in the endocervix^12–14^. AUB is characterized by changes in menstruation involving increased volume, duration, or frequency of bleeding, affecting 10%–30% of women of reproductive age^15–19^. Vaginitis accounts for 90% of cases caused by bacterial vaginosis (BV), Candida vaginitis (VVC), or Trichomonas vaginalis (TV)^20–25^.

These infections are associated with adverse outcomes such as preterm labor and delivery, pelvic inflammatory disease (PID), and postabortal endometritis^26–28^. Other diseases including other infections than HPV such as Neisseria gonorrhoeae and Chlamydia trachomatis, as well as atrophic vaginitis (AV), a condition affecting 25–50% of postmenopausal women, is another common affliction^29–31^. In addition to these diseases, other frequently encountered gynecologic conditions include uterine fibroids, endometrial polyps, and ovarian cysts, induced abortion, urinary tract infections (UTIs), cervical lesion treatments, and abnormalities detected via thin-prep cytologic test (TCT)^26–28,32–34^.

The vaginal microbiota (VMB) refers to the community of microorganisms present in the vaginal environment of women of reproductive age^35–39^. Around 2/3 of the healthy VMB is dominated by one of the *Lactobacillus* species, including *Lactobacillus crispatus*, *Lactobacillus iners*, *Lactobacillus gasseri*, and others^40–44^. These bacteria metabolize glycogen to produce lactic acid, which maintains the vaginal environment at an acidic pH^45–50^. Research has demonstrated that the VMB plays a critical role in the female vaginal microenvironment by supporting vaginal health and serving as the defense system against sexually transmitted infections (STIs)^51–55^.

Abnormal changes in the vaginal microenvironment, often characterized by a predominance of non–*Lactobacillus* species, result in the secretion of short-chain fatty acids (SCFAs) that compromise the protective mechanisms of the VMB^56–58^. Such changes disrupt the balance of the VMB and increasing the diversity of other microorganisms such as *Gardnerella vaginalis, Prevotella spp*., and *Sneathia spp*.^59–62^. This condition further damages the cervical epithelial barrier and facilitates the development of various diseases^63–66^. A non–*Lactobacillus* dominated vaginal microbiome, as observed in BV, is linked to a heightened risk of upper reproductive tract infections, yeast infections, miscarriage, preterm birth, UTIs, as well as sexual transmitted infections including HPV infection, and HIV infection^67–74^.

The immune system plays a critical role in cervical and vaginal health^75–78^. Persistent human papillomavirus (HPV) infection disrupts this balance, inducing chronic inflammation through the recruitment of various cytokines, such as GM-CSF, IFN-γ, IL-1α, IL-1β, IL-4, IL-6, IL-8, IL-10, IL-17A, MCP-1, MIG, RANTES, and TNF^79–81^. Cervical cancer further exacerbates this inflammatory response by activating immune cells via Toll-like receptors (TLRs), leading to the secretion of pro-inflammatory cytokines^53^. Among these, IL-6, IL-8, IL-4, and IL-10 have been identified as potential biomarkers for assessing invasive cancer and metastasis risk^53,81^. Elevated levels of IL-6, IL-17, and IL-8 are strongly associated with tumor progression^74,81^, while IL-1β, IL-4, IL-6, IL-10, and TNF-α correlate with increased susceptibility to cervical cancer^82–84^. The presence of IL-1β, IL-8, IL-6, and TNF-α indicates a severe chronic inflammatory state, which not only sustains HPV persistence but also accelerates cervical carcinogenesis^82–84^. Additionally, IL-1α and IL-1β stimulate fibroblast growth factor (FGF-7) upregulation in fibroblasts, promoting epithelial cell proliferation and tissue remodeling^84,85^. This process further facilitates angiogenesis and tumor progression^86,87^.

However, studies investigating immune responses in patients with common gynecologic diseases are scarce, especially among Chinese women^88–91^. To evaluate the expression patterns of these factors across different gynecological diseases and explore their potential relationships, we collected vaginal swabs from 310 patients with various gynecological diseases and 112 matched healthy volunteers. We further analyzed their vaginal microbiome and associated cytokine expression and evaluated their interactions both in silico and in vitro. The VMB results for HPV and CIN samples can be found in our earlier study^92^. This article includes additional vaginal microbiome data of five other gynecological conditions, including POP, CP, AUB, and various forms of vaginitis in gynecology clinics. Our study aimed to address this gap by focusing on the relationship between common gynecologic diseases in China, the VMB, and the immune system.

## Results

### Participant characteristics

After excluding patients’ samples with unclear diagnosis or missing information, 422 valid samples were included in the study. These participants were divided into eight groups: a healthy control group consisting of participants undergoing regular gynecological check-ups (*n* = 112), an HPV-positive group (*n* = 156), a CIN-diagnosed group (*n* = 97), a pelvic organ prolapse (POP) group (*n* = 9), a cervical polyp (CP) group (*n* = 6), an abnormal uterine bleeding (AUB) group (*n* = 8), a vaginitis group (*n* = 20), and an “other” group, which included participants with various other diseases (*n* = 14) (Supplementary Fig. 1 and Table 1).

**Table 1:** Basic information of clinical patients

	*n*	Age (Average)	Treatment
**Healthy Control (HC)**	112	25–64 (35)	-
**HPV-Positive (HPV)**	156	26–66 (39.2)	Baofukang Suppository (Hainan bikai Pharmaceutical Co., Ltd., China)
**CIN-Diagnosed (CIN)**	97	23–69 (39.4)	Loop Electrosurgical Excision Procedure (LEEP)
**Pelvic Organ Prolapse (POP)**	9	27–35 (31.6)	Electrical Stimulation Therapy
**Cervical Polyp (CP)**	6	32–50 (38.3)	Outpatient Surgical Treatment
**Abnormal Uterine Bleeding (ABU)**	8	26–51 (41.1)	Progesterone Treatment
**Vaginitis**	20	23–67 (37.8)	Antibiotic Treatment
**Other diseases**	14	25–60 (38.9)	Mix

## VMB diversity among different diseases

We did not observe significant difference among HC, HPV and CIN groups, although there was a trend with lower *L*. *crispatus* in HPV and CIN groups (Supplementary Fig. 2A). In beta-diversity analysis, the HPV, CIN and vaginitis groups showed a significantly different vaginal microbiome compared to the healthy control (Supplementary Fig. 2D, 3C). Among the small-sample experimental groups, such as POP, AUB, CP, vaginitis, and other groups, POP was a rather special group, as it had the highest species diversity, a lower percentage of *L*. *crispatus* and *L*. *iners*, and higher Simpson diversity index values compared to the HC group (Supplementary Fig. 2A–C). Since the number of cases in the POP, AUB, CP, vaginitis, and other groups was small, our analysis for these groups was only exploratory (Supplementary Figs. 2E, 3A–C). To avoid unintended overinterpretation, our study focused on the HPV and CIN groups in the following analysis (Supplementary Figs. 2D, 3D).

**Fig. 1:**
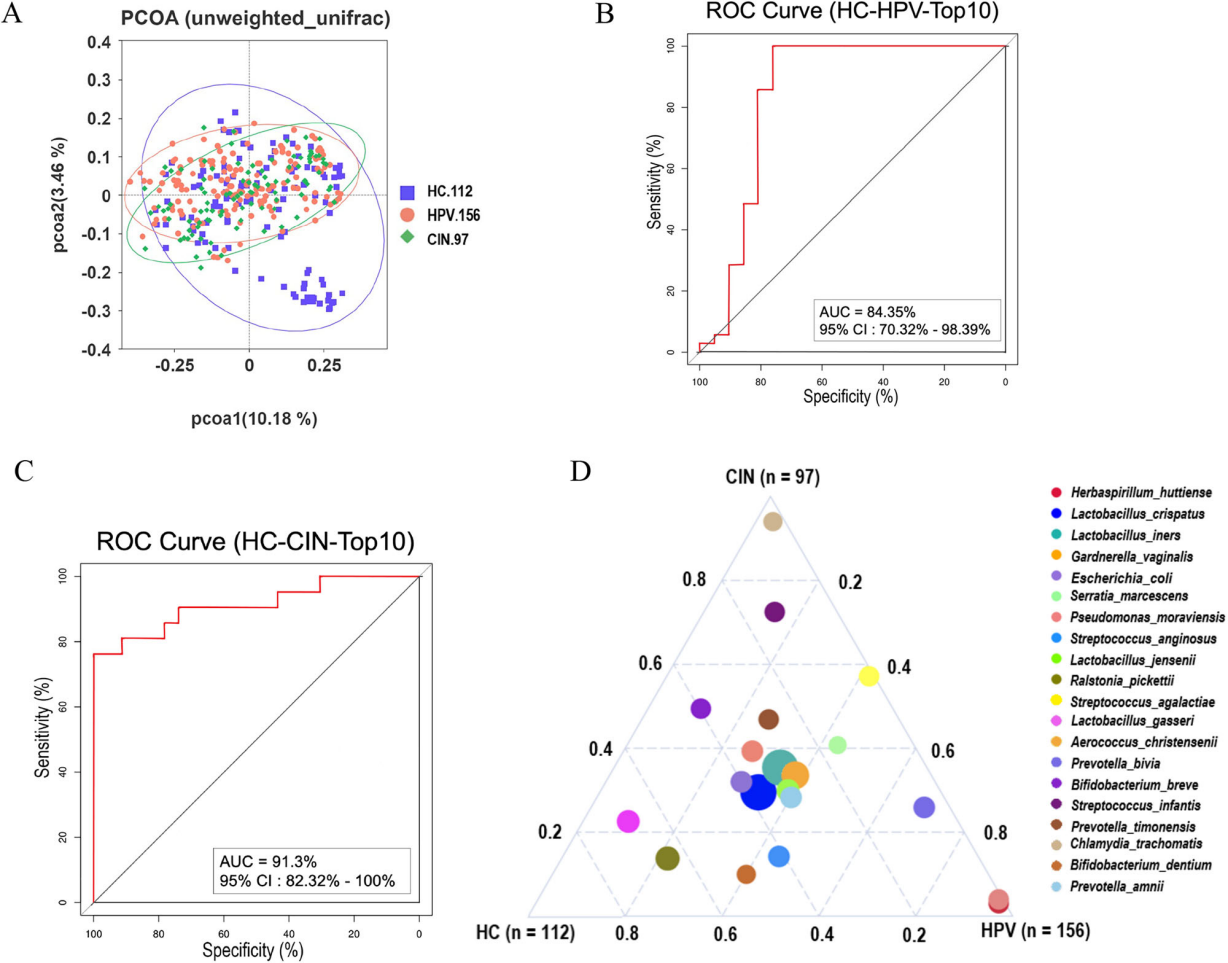
VMB of all the participants. **A** Beta diversity analysis of the OUT among the HC, HPV, and CIN groups with unweighted unifrac distance algorithm presented by PCoA (Principal Coordinates Analisis). **B** ROC (Receiver Operating Characteristic) curve when compared HPV groups and HC group with top ten distinct bacterial species. **C** ROC curve when compared CIN groups and HC group with top ten distinct bacterial species. **D** Terneryplot analysis of top bacterial species among the HC, HPV, and CIN groups.

We performed a random forest analysis on the HC, HPV, and CIN groups and identified the top ten species based on the Mean Decrease Accuracy (Supplementary Fig. 3E, F). Subsequently, we constructed Receiver Operating Characteristic (ROC) curves using cross-validation. The AUC reached 84.35% for the HC vs. HPV comparison (Fig. 1B), and 91.3% for the HC vs. CIN comparison (Fig. 1C). To understand the microbial distribution across the HC, HPV, and CIN groups, we analyzed the top 20 species by average abundance and visualized the data using a ternary plot (Fig. 1D). The enriched species were *L*. *gasseri* and *Ralstonia pickettii* in the HC group, *Bifidobacterium dentium* and *Escherichia coli* in the HPV group, *Bifidobacterium breve* and *Chlamydia trachomatis* in the CIN group. Other species, such as *L*. *crispatus*, *L*. *iners*, and *G*. *vaginalis*, were positioned in intermediate regions, indicating a shared distribution across the three groups (Fig. 1D).

### Cytokines among the HC, HPV, and CIN Groups

To further explore the interaction of vaginal microbiome and immune response, we had designed a study to measure 13 immune-related cytokines on 100 randomly selected clinical samples (HC group: *n* = 23; HPV group: *n* = 46; CIN group: *n* = 31) according to our previously researches: GM-CSF, IFN-γ, IL-1α, IL-1β, IL-4, IL-6, IL-8, IL-10, IL-17A, MCP-1, MIG, RANTES, and TNF. The data showed seven cytokines (GM-CSF, IFN-γ, IL-4, IL-10, IL-17A, RANTES, and TNF) did not show significant changes (Supplementary Fig. 4). Only IL-1α, IL-1β, IL-6, IL-8, MCP-1, and MIG could be reliably quantified from our clinical samples. Based on these findings, we proceeded to measure the concentrations of these six cytokines in the remaining samples in this study and analyzed their relationships with VMB composition and expression.

**Fig. 2:**
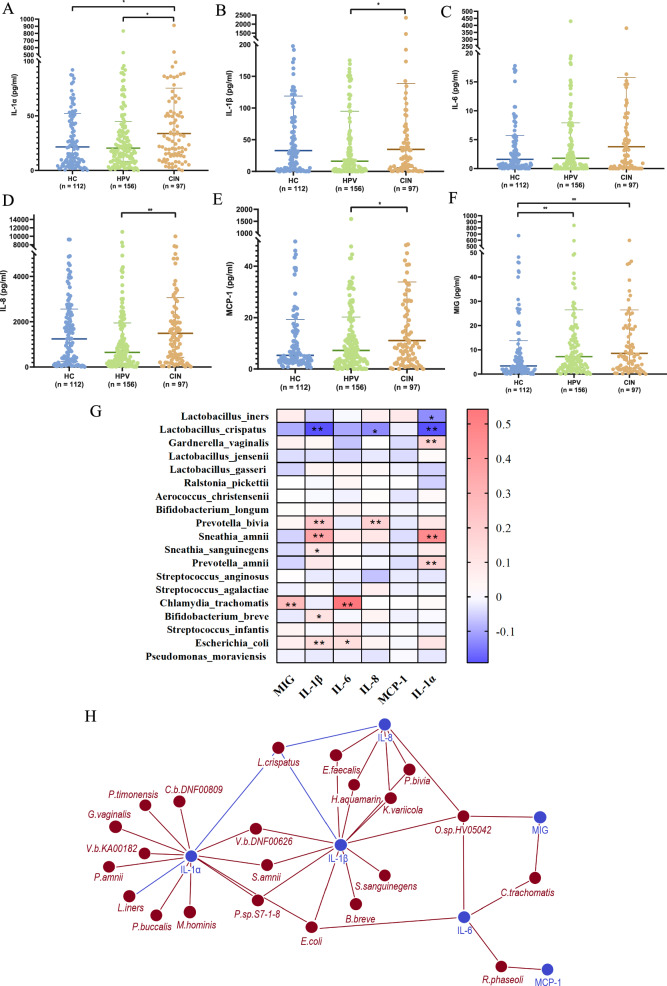
Cytokines and bacteria abundance among the HC, HPV and CIN groups. Concentrations of **A**) IL-1α (p_(CON-CIN_): 0.014, p_(HPV-CIN_): 0.011), **B** IL-1β (p_(HPV-CIN_): 0.017), **C** IL-6, **D** IL-8 (p_(HPV-CIN_)_:_ 0.004), **E** MCP-1 (monocyte chemoattractant protein) (p_(HPV-CIN_): 0.04) and **F** MIG (monokine induced by gamma-interferon) (p_(CON-HPV_): 0.003, p_(CON-CIN_): 0.004) among the HC, HPV and CIN groups measured by Cytometric Bead Array (CBA). (Fig. 2A–F used Kruskal Wallis analysis and Benjamini-Hochberg correction, p value has been adjusted, *p < 0.05; **p < 0.01; ***p < 0.001) **G** Correlation of the six tested cytokines and the top 19 strains from vaginal microbiome data presented by heatmap. **H** Predicted network of the top 50 bacteria which have statistic significance association with the tested cytokines. (Fig. 2G, H used Pearson analysis, *p < 0.05; **p < 0.001).

Among these, MIG and IL-8 showed significant differences across the eight disease groups (Supplementary Fig. 5A–F). Due to the much larger sample sizes in the HC, HPV, and CIN groups compared to the other five groups, we focused on these three groups for further analysis. Compared with the healthy group, IL-1α and MIG concentration was significantly higher in HPV and CIN groups (Fig. 2A–F). In addition, concentrations of IL-1α, IL-1β, IL-8, and MCP-1 were significantly elevated in the CIN group compared to the HPV group (Fig. 2A–F).

To further explore single and multiple HPV infections, we compared the healthy control group (HC group), the single HPV infection group (HPV-S group), the multiple HPV infection group (HPV-M group), and the single and multiple infection CIN group (Supplementary Fig. 6A–F). We found that the expression of MIG in both the HPV-S and HPV-M infection CIN groups, as well as in the HPV-M infection group, was significantly higher than in the healthy control group (Supplementary Fig. 6F). The expression of IL-8 in the HPV multiple infection CIN group was significantly higher than that in the HPV multiple infection group (Supplementary Fig. 6D). We also analyzed the expression of six selected cytokines in different HPV single infection genotypes, including genotypes 16, 18, 42, 44, 52, 53, 58, and 81 (Supplementary Fig. 7A–F). No significant differences were observed when these genotypes were compared with the healthy control group.

We further investigated the correlation between the six selected cytokine expression and the top 19 bacterial species (Fig. 2G). MIG expression was positively related to *C*. *trachomatis* only. IL-1β was negatively related to *L*. *crispatus* but positively correlated to *B*. *breve, Sneathia amnii, Sneathia sanguinegens, Prevotella bivia* and *E*. *coli. E*. *coli* and *C*. *trachomatis* have a positive relationship with IL-6. *P*. *bivia* was positively correlated with IL-8, while *L*. *crispatus* has a negative relationship with IL-8. *G*. *vaginalis, S*. *amnii*, and *Prevotella amnii* were positively correlated with IL-1α, while *L*. *crispatus* and *L*. *iners* have negatively correlated (Fig. 2G). Their correlations were also presented in the network with the red lines showed the positive correlation, and blue lines demonstrated the negative correlation (Fig. 2H).

### Cytokines according to VMB

**Fig. 3:**
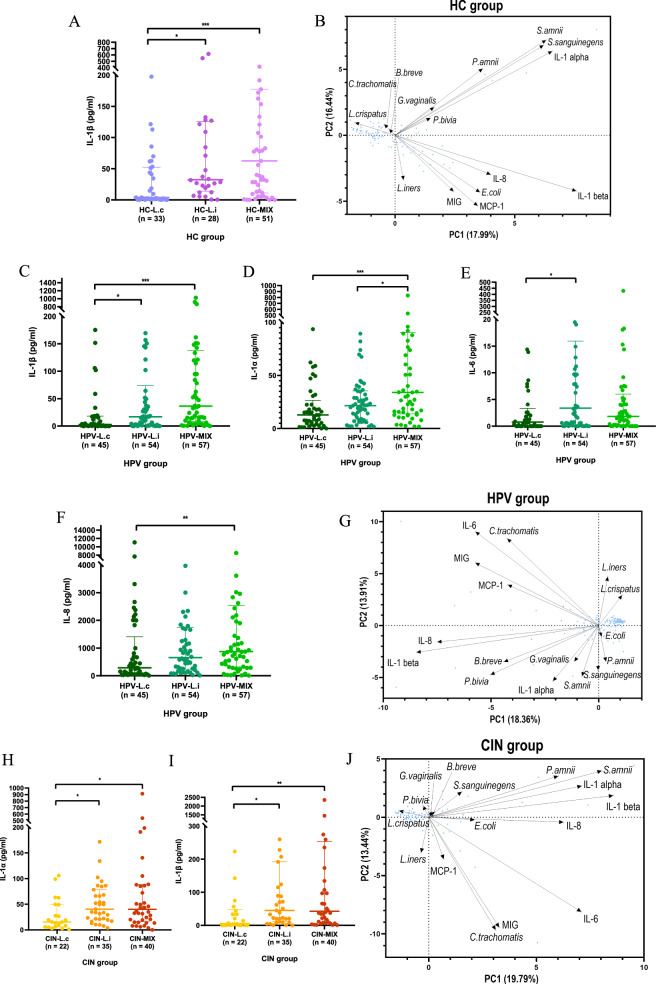
Correlation of cytokines and subgroups of the healthy, HPV and CIN groups. **A** IL-1β expression level among *L*. *crispatus* (*L.c*) dominated group (*n* = 33), *L*. *iners* (*L.i*) dominated group (*n* = 28), and non-*Lactobacillus* dominated (mix) subgroups (*n* = 51) in healthy participants (p(_*L.c-L.i*_)*:* 0.017, p(_*L.c*__-MIX_): 0.001). **B** The correlation of top bacterial species and cytokines in healthy participants presented by principal component analysis (PCA). **C**–**F** Expression level of IL-1α (p_(*L.c-*MIX_): < 0.001, p_(*L.i*-MIX_): 0.043), IL-1β (p_(*L.c-L.i*_)*:* 0.048, p_*(L.c*-MIX_): _<_ 0.001), IL-6 (p_(*L.c-L.i*_): 0.038) and IL-8 (p_(*L.c*-MIX_): 0.006) among *L. crispatus* (*L.c*) dominated group (*n* = 45), *L*. *iners* (*L.i*) group (*n* = 54), and non-*Lactobacillus* dominated (mix) subgroups (*n* = 57) among HPV infected individuals. **G** The correlation of top bacterial species and cytokines among HPV infected individuals presented by PCA. **H**, **I** Expression level of IL-1α (p_(*L.c-L.i*_): 0.022, p_(*L.c*-MIX_): 0.018) and IL-1β (p_(*L.c-L.i*_): 0.022, p_(*L.c*-MIX_): 0.006) among *L*. *crispatus* (*L.c*) dominated group (*n* = 22), *L*. *iners* (*L.i*) dominated group (*n* = 35), and non-*Lactobacillus* dominated (mix) subgroups (*n* = 40) among the CIN patients. **J** The correlation of top bacterial species and cytokines among CIN patients presented by PCA. (Fig. 3A, C–F, H, and I used Kruskal Wallis analysis, and Benjamini-Hochberg correction, p value has been adjusted *p < 0.05; **p < 0.01; ***p < 0.001).

To explore the correlation between cytokines and the vaginal microbiome under different disease conditions, we divided the HC, HPV, and CIN groups into three subgroups based on the dominant bacterial species: *L*. *crispatus*-dominated, *L*. *iners*-dominated, and MIX (*Non-Lactobacillus*-dominated). We then compared cytokine expression among these subgroups within the same condition. In the HC group, the *L*. *iners*-dominated (*n* = 28) and MIX (*n* = 51) sub-groups exhibited significantly higher IL-1β levels than the *L*. *crispatus*-dominated (*n* = 33) subgroup (Fig. 3A). No other cytokines showed significant differences between the subgroups (Supplementary Fig. 8A–E). The PCA plot indicated a correlation between IL-1β and bacterial species such as *L*. iners and *E*. *coli*, as these strains clustered in the same quadrant and direction as IL-1β (Fig. 3B).

In the HPV group, the MIX subgroup (*n* = 57) had significantly higher concentrations of IL-1α, IL-1β, and IL-8 compared to the *L*. *crispatus*-dominated (*n* = 45) subgroup (Fig. 3C, D, and F). In addition, IL-1β and IL-6 concentrations were significantly higher in the *L*. *iners*-dominated (*n* = 54) subgroup compared to the *L*. *crispatus*-dominated subgroup (Fig. 3D, E). Additionally, the concentration of IL-1α is higher in the MIX subgroup than the *L*. *iners*-dominated subgroup (Fig. 3C). No significant differences were found in the expression of MCP-1 and MIG across these three subgroups (Supplementary Fig. 8F, G). In the PCA plot, IL-6 clustered with *C*. *trachomatis*, while IL-1α, IL-1β, and IL-8 were associated with species such as *B*. *breve, P*. *bivia, G*. *vaginalis*, and *S*. *amnii* (Fig. 3G).

In the CIN group, the *L*. *iners*-dominated (*n* = 35) and MIX (*n* = 40) sub-groups showed significantly higher concentrations of IL-1α and IL-1β compared to the *L*. *crispatus*-dominated (*n* = 22) subgroup (Fig. 3H, I). No significant changes were observed in IL-6, IL-8, MCP-1, or MIG (Supplementary Fig. 8H–K). The PCA plot indicated that *B*. *breve, G*. *vaginalis, S*. *amnii, S*. *sanguinegens*, and *P. amnii* were associated with IL-1α and IL-1β (Fig. 3J).

By analyzing cytokine concentrations within the same subgroups across the three health and disease groups, we found no significant differences in cytokine levels among the HC, HPV, and CIN subgroups in the *L*. *crispatus*-dominated and MIX subgroups (Supplementary Fig. 9). However, in the *L*. *iners*-dominated subgroup, IL-1α and IL-8 concentrations were significantly higher in CIN patients compared to HPV-infected individuals (Supplementary Fig. 10A, B). Additionally, MIG expression was elevated in *L*. *iners*-dominated CIN patients compared to *L*. *iners*-dominated healthy individuals (Supplementary Fig. 10C). These findings suggest that although similar inflammatory cytokines were elevated in CIN patients compared to healthy and/or HPV-infected individuals, the microbiome associated with these cytokines may differ.

### Interaction networks of bacterial species and cytokines

**Fig. 4:**
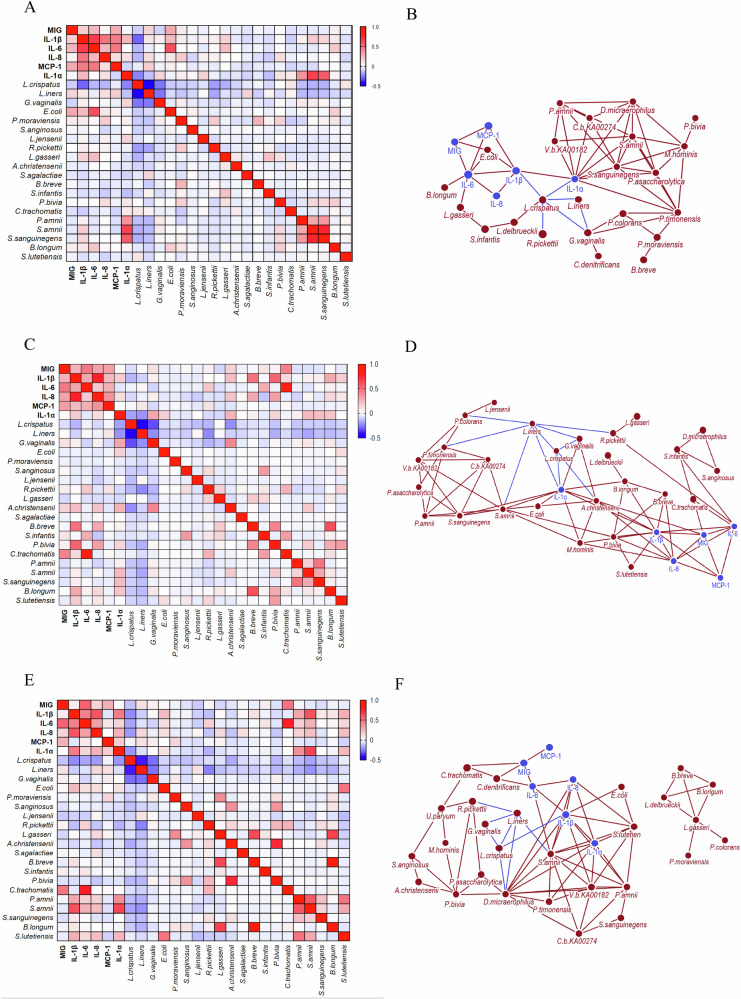
Pearson Heatmap and network in HC, HPV and CIN groups. **A** Heatmap of the six tested cytokines and top 20 strains in the HC group. **B** Network of top 30 bacterial species, which have statistic significant with related cytokines in HC group. **C** Heatmap of the six tested cytokines and top 20 bacterial species, which have statistic significant with related cytokines in the HPV group. **D** Network of top 30 bacteria which have statistic significant with related cytokines in HPV group. **E** Heatmap of the six tested cytokines and top 20 strains in the CIN group. **F** Network of top 30 bacteria which have statistic significant with related cytokines in the CIN group. (Fig. 4A–F used Pearson analysis, *p < 0.05; **p < 0.001).

We also evaluated the correlation and network considering both the bacterial species and cytokines within the HC, HPV, and CIN groups. In the HC group, *L*. *crispatus* showed a significant negative correlation with IL-1α, IL-1β, as well as with *L*. *iners* and *G*. *vaginalis* (Fig. 4B). IL-1α exhibited a significantly positive correlation with *P*. *amnii, S*. *amnii*, and *S*. *sanguinegens*, which formed an interactive network. Additionally, IL-6, IL-1β and MIG were positively correlated with *E*. *coli* (Fig. 4A, B).

In the HPV group, IL-1α showed a significant negative correlation with *L*. *crispatus* and *L*. *iners* (Fig. 4D) and a positive correlation with *G*. *vaginalis, E*. *coli, Aerococcus christensenii, S*. *amnii*, *S*. *sanguinegens*, and *Bifidobacterium longum*. IL-8 exhibited a significant positive correlation with *A*. *christensenii, B*. *breve*, and *P*. *bivia*. Additionally, IL-6 showed a strong positive association with *Ralstonia pickettii*, *Streptococcus infantis*, and *C*. *trachomatis*, while IL-1β was positively correlation with *A*. *christensenii, B*. *breve, P*. *bivia, B*. *longum*, and *Streptococcus lutetiensis*. Notably, *L*. *iners* was a key bacterial species in the network, showing a negative correlation with multiple species, including *L*. *crispatus*, *G*. *vaginalis* and *S*. *amnii* (Fig. 4C, D).

In the CIN group, several bacteria species showed a strong correlation with IL-1β, including *E*. *coli, P*. *amnii, S*. *amni*i, and *S*. *lutetiensis* (Fig. 4F). Notably, only *L*. *crispatus* exhibited a strong negative correlation with different cytokines. IL-1α was positively correlated with *P*. *amnii, S*. *amni*i, and *S*. *lutetiensis*, while IL-8 showed a significant positive correlation with *S*. *amnii* and *S*. *lutetiensis*. Furthermore, *S*. *amnii, Dialister micraerophilus, and P*. *amnii* formed a positively interactive network, potentially supporting each other and contributing to the production of IL-1α and IL-1β, IL-6 and IL-8 (Fig. 4E, F).

### Cytokine production after co-culturing with different vaginal bacterial species

To validate whether bacteria contribute to cytokine changes, we co-cultured HeLa cells with various bacterial species that were significantly correlated with cytokine levels and measured the corresponding cytokine concentrations (IL-1α, IL-1β, IL-6, and IL-8) in the supernatant after 6 and 24 hours in vitro. At 6 hours, *G*. *vaginalis*, *P*. *bivia*, *S*. *amnii*, *S*. *sanguinegens*, and *B*. breve significantly increased IL-1β concentrations, while *G*. *vaginalis*, *P*. *bivia, S*. *amnii, B*. *breve*, and *Fannyhessea vaginae* significantly elevated IL-1α levels. Furthermore, *L*. *gasseri* led to a decrease in IL-1β after 24 hours of co-culture.

**Fig. 5:**
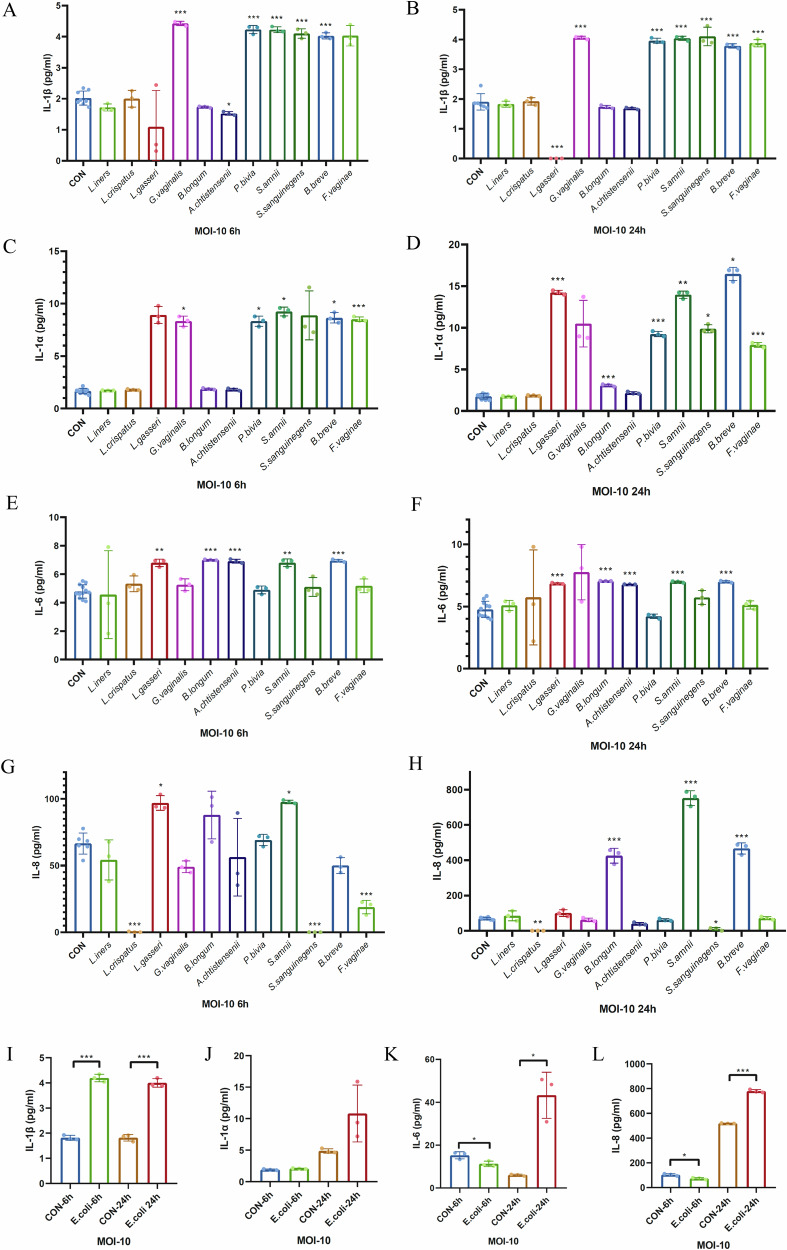
Cytokines concentration tested by ELISA after co-culture with Hela cells. **A**, **B** IL-1β concentration after Hela cells co-cultured with different bacterial species for 6 **A** (p_(*G.vaginalis*_): < 0.001, p_(*A.chtistensenii*_): 0.021, p_(*P.bivia*_): _<_0.001, p_(*S.amnii*_): < 0.001, p_(*S.sanguinegens*_): < 0.001, p_(*B.breve*_): < 0.001) and 24 **B** hours (p_(*L.gasseri*_): < 0.001, p_(*G.vaginalis*_): < 0.001, p_(*P.bivia*_): < 0.001_,_ p_(*S.amnii*_): < 0.001, p_(*S.sanguinegens*_): < 0.001, p_(*B.breve*_)_:_ < 0.001_,_ p_(*F.vaginae*_): < 0.001). **C**, **D** IL-1α concentration after Hela cells co-cultured with different bacterial species for 6 (C) _(_p_(*G.vaginalis*_): 0.049, p_(*P.bivia*_): 0.049, p_(*S.amnii*_): 0.02, p_(*B.breve*_): 0.049, p_(*F.vaginae*_):< 0.001) and 24 (D) hours (p_(*L.gasseri*_): _<_ 0.001, p_(*B.longum*)_: < 0.001, p_(*P.bivia*_): < 0.001_,_ p_(*S.amnii*_): 0.003_,_ p_(*S.sanguinegens*_): 0.019, p_(*B.breve*_): 0.035, p_(*F.vaginae*_): _<_ 0.001). **E**, **F** IL-6 concentration after Hela cells co-cultured with different bacterial species for 6 (E) _(_p_(*L.gasseri*_): 0.004, p_(*B.longum*_): < 0_._001, p_(*A.chtistensenii*)_: < 0.001, p_(*S.amnii*_): 0.005, p_(*B.breve*_): < 0.001) and 24 (F) hours (p_(*L.gasseri*_): < 0.001, p_(*B.longum*_): < 0.001, p_(*A.chtistensenii*_): < 0_._001, p_(*S.amnii*_): < 0.001, p_(*B.breve*_): < 0.001). **G**, **H** IL-8 concentration after Hela cells co_-_cultured with different bacterial species for 6 (G) (p_(*L.crispatus*_): < 0.001, p_(*L.gasseri*_): 0_._042, p_(*S.amnii*_): 0.032, p_(*S.sanguinegens*_): < 0.001_,_ p_(*F.vaginae*_):< 0.001) and 24 _(_H) hours (p_(*L.crispatus*_): 0.005_,_ p_(*B.longum*_): < 0.001, p_(*S.amnii*_): < 0.001, p_(*S.sanguinegens*_): 0.027, p_(*B.breve*_): < 0.001). **I–L**) Concentration of IL-1β _(_I) (6 h, p_(CON-*E.coli*_): < 0.001; 24 h, p_(CON-*E.coli*_): < 0.001), IL_-_1α (J), IL_-_6 (K) (6 h, p_(CON-*E.coli*_): 0.034; 24 h, p_(CON-*E.coli*_): 0.027), and IL-8 (L) (6 h, p_(CON-*E.coli*_): 0.013; 24 h, p_(CON-*E.coli*_): _<_ 0.001) in the supernatant after Hela cells co-cultured with *E*. *coli* for 6 and 24 hours. Figs. 5A–C, 5E–G, and D, H used One-way ANOVA analysis, *p < 0.05; **p < 0.01; ***p < 0.001; Fig. 5I–L used T-test analysis, *p < 0.05; **p < 0.01; ***p < 0.001).

*L*. *crispatus* and *S*. *sanguinegens* exhibited lower IL-8 expression compared to the control condition, whereas *S*. *amnii* increased IL-8 levels as early as 6 hours of co-culture. Additionally, *L*. *gasseri, B*. *longum, A*. *christensenii, S*. *amnii*, and *B*. *breve* significantly increased IL-6 concentrations at both 6 and 24 hours (Fig. 5A–H). For *E*. *coli*, which was co-cultured under aerobic conditions, all cytokine concentrations were significantly higher compared to the control condition at 24 hours (Fig. 5I–L).

We also performed co-culture experiments using VK2 cells, an immortalized human vaginal epithelial cell line. Based on our previous experiments with HeLa cells, we selected *L*. *crispatus*, *L*. *iners*, *G*. *vaginalis*, and *P*. *bivia* for co-culture with VK2 cells and measured cytokine levels at 6 and 24 hours (Supplementary Fig. 11). The concentration of IL-1β in the *G*. *vaginalis* group was significantly higher than in the control group at both time points. Similarly, the expression of IL-1α and IL-6 was markedly elevated after 24 hours, and IL-8 was significantly higher at both 6 and 24 hours. In contrast, cytokine levels in the *L*. *crispatus* and *L*. *iners* groups showed no significant change. The overall trend across bacterial groups was largely consistent with the results from HeLa cells, with minor differences. Despite the known limitations of HeLa cells, the concordant results from VK2 cells strengthen the conclusion that non-*Lactobacillus* bacteria promote the secretion of pro-inflammatory cytokines.

In summary, bacterial species had varying effects on cytokine expression. Some bacteria, such as *G*. *vaginalis, S*. *amnii*, and *F*. *vaginae*, increased the levels of IL-1β, IL-1α, and IL-8, while others, like *L*. *crispatus*, decreased IL-8 levels. These patterns observed in the in vitro co-culture experiments closely resembled the data from the clinical samples, suggesting that the bacteria present in the vaginal microbiome influence immune responses, which may contribute to gynecological diseases.

## Discussion

In this study, we collected vaginal swab samples from 422 individuals, including healthy controls and patients diagnosed with HPV infection, cervical intraepithelial neoplasia (CIN 1, 2, and 3), pelvic organ prolapse (POP), chronic pelvic pain (CP), abnormal uterine bleeding (AUB), vaginitis, and other gynecological disorders (Table 1). We presented the correlation between VMB and associated cytokines, especially focusing on BV-related bacteria and inflammatory cytokines, and validated our results through in vitro co-culture experiments.

We quantified 13 cytokines and observed significant differences in diversity (Supplementary Figs. 2D, 3C) and expression levels of IL-1α, IL-1β, IL-6, IL-8, MCP-1, and MIG among HPV-infected and CIN patients compared to healthy individuals (Fig. 4A–F). The CIN group exhibited markedly higher concentrations of pro-inflammatory cytokines compared to the HPV-infected and HC groups (Fig. 2A–F), indicating a heightened inflammatory microenvironment in CIN patients. These data are supported by other studies showing that persistent HPV infection induces chronic inflammation, mediated by a cascade of cytokines such as GM-CSF, IFN-γ, IL-1α, IL-1β, IL-4, IL-6, IL-8, IL-10, IL-17A, MCP-1, MIG, RANTES, and TNF^79,80,93,94^. Similarly, IL-6, IL-8, IL-1α, and IL-1β serve as potential biomarkers for cervical cancer progression and metastasis, reflecting a pronounced chronic inflammatory state^81–83,94^. Elevated levels of IL-8 and IL-1β are associated with enhanced HPV persistence and CIN progression^82–84^, while IL-6 overexpression correlates with poorer cervical cancer prognosis^95,96^.

Among the HPV-positive donors, cases of both single and multiple genotype infections were observed. Single infections were categorized as either high-risk or low-risk HPV. Multiple infections included combinations of high-risk genotypes, high-risk and low-risk genotypes, and others, as detailed in our prior work^92^. Our observations showing elevated MIG expression in HPV multiple infection and CIN patients, along with higher IL-8 levels specifically in the HPV multiple infection CIN group, suggest a weak correlation between HPV genotype and inflammatory cytokine levels (Supplementary Fig. 6D, F). Notably, the interaction between cytokines and the vaginal microbiome appears stronger and more aligned with disease progression stages (Figs. 2G, 3A–J, Supplementary Figs. 5A–F, 6A–F).

Confounding factors for HPV infection and CIN diagnosis have been observed and published in our previous study^92^. We collected potential risk factors from patients to analyze using logistic regression, including age, CST types, pregnancy, gestation, number of sexual partners, smoking, vaginal douching, contraception, and IUD implantation, among others^92^. We found that age, CST types, pregnancy, number of sexual partners, contraception, and IUD implantation were risk factors for HPV infection^92^. For CIN diagnosis, only HPV infection was identified as a risk factor^92^. However, regarding the correlation between cytokines and the vaginal microbiome, we did not account for potential confounders, which is a limitation of this study and should be considered when interpreting the data.

When we further evaluated the correlation between disease-related cytokines and the vaginal microbiome, we found that *Lactobacillus* spp., especially *L*. *crispatus* exhibited negative correlations with pro-inflammatory cytokines, suggesting that these species may potentially play a role in inhibiting inflammation. This aligns with other research showing that *Lactobacillus*-dominated vaginal microbiome, which metabolizes glycogen into lactic acid to maintain a low vaginal pH, suppresses non-*Lactobacillus* bacteria and HPV infection while also modulating inflammation to preserve vaginal homeostasis^83,97^.

In vaginosis conditions, elevated levels of short-chain fatty acids (SCFAs) have been linked to increased inflammation^66,98^, creating a favorable environment for anaerobic bacteria. These bacteria produce virulence factors that degrade mucin, compromise epithelial barrier integrity, and trigger pro-inflammatory responses^61^. Consistent with prior research, our study identified a positive correlation between non-*Lactobacillus* species and inflammatory cytokines, reinforcing the role of dysbiosis in promoting inflammation. Specifically, *B*. *breve*, *P*. *bivia*, *G*. *vaginalis*, *S*. *amnii*, *S*. *sanguinegens*, *P*. *amnii*, *E*. *coli*, and *C*. *trachomatis*, were strongly associated with elevated levels of inflammation-related cytokines, suggesting their potential contribution to a pro-inflammatory vaginal microenvironment (Fig. 2G, H).

*L*. *iners* is a peculiar member of the *Lactobacilli* as it does not secrete lactic acid, implying a reduced protective effect on vaginal epithelial cells compared to other *Lactobacilli*. For this reason, the CST-III (*L*. *iners*-dominated) profile is frequently interpreted as a transitional state that is unstable and may either revert to a healthy CST (I, II, or V) or deteriorate into the dysbiotic CST-IV. The overall role of *L*. *iners* is therefore complex and not yet definitively understood. Some evidence suggests it may have a neutral or beneficial function, potentially acting as a pioneer species that can be leveraged therapeutically to prevent disease progression and steer the microbiota toward a healthy composition. Due to the complex role of *L*. *iners* in the vaginal microbiome and immune response, we further stratification into three subgroups, *L*. *crispatus*-dominated, *L*. *iners*-dominated, and non-*Lactobacillus*-dominated (MIX). We revealed that both *L*. *iners*-dominated and MIX subgroups were associated with elevated inflammation, regardless of whether samples came from healthy controls (HC), HPV-infected, or CIN groups. This suggests that *L*. *iners* may exhibit pro-inflammatory effects similar to non-*Lactobacillus* bacteria to some degree.

Our network analysis, which integrated both the vaginal microbiome and cytokines, revealed that IL-1α and IL-1β, cytokines that promote epithelial cell proliferation and tissue development, further driving angiogenesis and tumor progression, correlate with a cluster of vaginal bacterial species, forming a robust interaction network (Figs. 2G, H)^84–87^. *L*. *crispatus* consistently showed negative correlations with various bacteria, suggesting a stable, anti-inflammatory role across all subgroups (HC, HPV, and CIN). Except for its negative interaction with IL-1α, *L*. *iners* primarily exhibited negative correlations with other vaginal species, including *L*. *crispatus* and non-*Lactobacillus* species such as *G*. *vaginalis*, *S*. *amnii*, and *Prevotella spp*., indicating a potential role in maintaining microbiome stability by suppressing the overgrowth of BV-related microbes. The distinct bacterial and cytokine networks observed across subgroups (Fig. 4) also suggest that similar inflammatory responses may be triggered by different bacterial species under varying health and disease conditions.

To validate the observed correlations from clinical samples, we further tested whether the microbes that significantly triggered or inhibited cytokine expression exhibited a similar trend when interacting with two cell lines. The in vitro co-culture experiments across a collection of tested bacterial species, including both *Lactobacillus* species and BV-related microbes, largely recapitulated the trends observed in clinical samples (Figs. 5E–H, 5K, and 5L). These experiments confirmed that non-*Lactobacillus* bacteria, especially *G*. *vaginalis*, *Sneathia spp*., *Prevotella spp*., and *E*. *coli*, promote the secretion of pro-inflammatory cytokines, including IL-6, IL-8, IL-1α, and IL-1β, whereas *L*. *crispatus* significantly suppressed the production of these cytokines, particularly IL-8. This consistency reinforces the reliability of our findings and aligns well with our clinical observations, supporting their biological relevance.

A key limitation of this cross-sectional study is its inability to determine causality. Although we have identified significant associations between vaginal microbial composition, local inflammatory cytokine levels, and disease status (HPV/CIN), the design captures data from only one time point in the integrated disease course. We collected vaginal secretion samples at a specific time point without tracking subsequent disease progression. As a cross-sectional study, this approach limits our ability to establish causality or observe temporal changes. Future work should aim to conduct longitudinal cohort studies that track donors over time, documenting how microbiota and cytokines evolve with disease progression or regression. In addition, the mechanisms regarding the detailed causality and signaling pathways and related gene expression still require further investigation.

Furthermore, in this study, bacterial communities of clinical samples were categorized based on 16S rRNA gene sequencing relative abundance as *L*. *crispatus*-dominated, *L*. *iners*-dominated, or non-*Lactobacillus*-dominated (MIX). The relationship between bacterial species and cytokines was also analyzed using relative abundance data. Previous research has shown that the total bacterial load is associated with the expression of inflammation-related cytokines, independent of relative microbial proportions^99^. Another limitation emerges when analyzing the relationship between bacteria and inflammatory cytokines using relative abundance rather than total bacterial load, as microbial load and composition are distinct variables. For example, even if one bacterial species has a higher relative abundance in a sample, its actual bacterial load may remain low if the total bacterial load is minimal. Encouragingly, the trends observed in our in vitro experiments, where bacterial load was standardized to ensure consistency across conditions, align well with clinical findings. It would be highly valuable for future studies to measure total bacterial load using alternative methods, such as qPCR or spike-in normalization, which we would aim to implement if possible. Moreover, our basic experiment, while showing trends consistent with clinical samples, has a limitation as it excludes immune cells, the vaginal mucus layer, and full tissue architecture, differing from the in vivo vaginal environment. Future efforts will focus on developing a 3D vaginal model or utilizing ex vivo explants to better mimic the internal vaginal environment and account for the influence of immune and tissue cells.

A further limitation is the unknown menstrual cycle stage of the participants at the time of sampling. Samples were collected during colposcopy appointments, where clinical protocol requires avoidance of menstruation to facilitate examination and procedures. Therefore, while we can confirm that no samples were collected during active menstruation, we lack data on the specific cycle phase (e.g., follicular, luteal). Since hormonal fluctuations across the menstrual cycle are known to influence both the VMB and cytokine expression, the menstrual phase is a potential confounding factor.

In summary, our research examined the correlation between the vaginal microbiome and cytokines using both clinical samples and in vitro experiments. We found higher alpha-diversity and a lower percentage of *Lactobacillus spp*. among disease groups. Additionally, we observed that both *L*. *crispatus* and *L*. *iners* were associated with decreased inflammatory cytokines, while non-*Lactobacillus* bacteria were linked to higher inflammatory cytokines. Our findings contribute to a deeper understanding of the role of VMB in gynecological health and inflammation, highlighting the potential application of *Lactobacillus spp*. for reducing inflammation associated with HPV-related diseases and other gynecological conditions.

## Methods

### Study Population

Participants were patients who visited the gynecological clinic at Aviation General Hospital of China Medical University from November 2020 to May 2021. Healthy volunteers were individuals who visited the same hospital for regular health screenings. The study was approved by the Aviation General Hospital Ethics Committee under the certificate number 2021-KY-01-02. The inclusion criteria for participants were as follows: (a) aged between 20 and 70 years; (b) willingness to participate in the study during the collection of cervical and vaginal samples for regular screening; (c) voluntary participation with signed written informed consent; and (d) no antibiotic use in the past one or two weeks. Exclusion criteria included: (a) pregnancy; (b) prior vaccination against HPV; (c) engagement in sexual activity or drug application (e.g. antibiotic treatment or Baofukang suppository application) within 48 hours before sampling; (d) diagnosis of tuberculosis, hepatitis B virus (HBV), hepatitis C virus (HCV), sexually transmitted infections other than HPV (e.g., HIV, *Treponema pallidum*); and (e) immune system disorders or chronic diseases such as diabetes mellitus, hypertension, or coronary heart disease.

### Sample collection and DNA extraction

Clinical samples were collected using two cervical swabs (Huaxia Ruitai Plastic Industry Company, China) inserted 2–3 cm into the vagina. One swab was rinsed in a vial containing normal saline solution for 16S rRNA gene sequencing, while the second swab was rinsed in a vial containing PBS for cytokine analysis. After collection, samples were immediately transferred to a − 80 °C freezer for storage until further analysis. DNA was extracted using the Fast DNA SPIN extraction kit (QIAamp PowerFecal Pro DNA Kit, Qiagen, Germany) following the standard protocol, yielding a final 100 μL of DNA, which was stored at −80 °C.

### 16S rRNA gene sequencing

16S rRNA gene sequencing was performed by NovoGene (Beijing, China). Briefly, all DNA samples were diluted to 1 ng/μL with sterile water. The V3–V4 hypervariable region of the 16S rRNA gene was amplified using primers 515 F/806 R with unique barcodes for each sample. PCR amplification was carried out using the Phusion High-Fidelity PCR Master Mix with GC Buffer (New England Biolabs, France) under optimized thermal cycling conditions to minimize amplification bias.

Amplicons were purified using AMPure XP beads (Beckman Coulter, USA) to remove primer dimers and unwanted fragments. Library preparation was performed using the TruSeq DNA PCR-Free Sample Preparation Kit (Illumina Inc., USA) to reduce PCR-induced bias and improve sequence accuracy. Library quality and concentration were assessed using a Qubit fluorometer (Thermo Fisher Scientific, UK) and an Agilent 2100 Bioanalyzer (Agilent Technologies, USA). The prepared libraries were then sequenced on the NovaSeq high-throughput sequencing platform (PE250, Illumina Inc., USA) to generate paired-end reads.

### Sequencing data analysis

After removing barcode and primer sequences, reads from each sample were merged using FLASH (v1.2.7) (http://ccb.jhu.edu/software/FLASH/) to generate the raw tags. These raw tags were subjected to stringent quality filtering to produce high-quality clean tags. The quality control process followed the reference Qiime Tags approach, and the final effective tags were obtained using the Uparse algorithm (http://drive5.com/uparse/). Sequences were clustered into operational taxonomic units (OTUs) at 97% identity by default. OTU sequences were annotated to the species level using the Mothur method and the SSUrRNA database (SILVA138). Multiple sequence alignment was performed using MUSCLE software (https://www.drive5.com/muscle/). Qiime software (v1.9.1) was employed to calculate OTUs and perform Chao1, Shannon, Simpson, and beta-diversity analyses. Additionally, R software (v2.15.3) was used to generate dilution curves, rank abundance curves, species accumulation curves, and Principal Coordinates Analysis (PCoA) plots.

### Cytometric Bead Array (CBA)

The concentrations of cytokines in samples were tested using the BD™ Cytometric Bead Array (Becton, Dickinson and Company, USA) following the standard protocol. Freeze-dried cytokine protein capture beads were reconstituted and added to the same test tube. The capture beads were then mixed and added to flow tubes. Equal amounts of both standard and sample solutions were added to each tube. The tubes were mixed well and incubated at room temperature, away from light, for one hour. After incubation, wash buffer was added to remove excess antibodies, and the antibody solution was resuspended for analysis using a cytometric bead array machine. Cytokine concentrations were measured using the FACSCanto II (R33896203057), and data analysis was performed using FCAP Array™ Software Version 3.0.1. Information of antibodies used in this study showed in supplementary table 1.

### Co-culture and supernatant collection

Hela cells were cultured in Dulbecco’s Modified Eagle’s Medium (DMEM) with high glucose (Sigma, Germany) and supplemented with 10% fetal bovine serum (FBS-HI) (Sigma, Germany) at 37°C with 5% CO_2_. VK2 cells were cultured in Keratinocyte SFM (serum-free medium) (Thermo Fisher Scientific, USA) with human recombinant epidermal growth factor (rEGF) (Thermo Fisher Scientific, USA) and bovine pituitary extract (BPE) (Thermo Fisher Scientific, USA) at 37°C with 5% CO_2_. Bacterial strains were purchased from the Culture Collection University of Gothenburg (CCUG, Sweden), including *Lactobacillus crispatus* (CCUG 38700), *Gardnerella vaginalis* (CCUG 44012), *Prevotella bivia* (CCUG 34046), *Lactobacillus iners* (CCUG 44108), *Bifidobacterium longum* (CCUG 59493), *Aerococcus christensenii* (CCUG 28831T), *Fannyhessea vaginae* “V” (CCUG 44116), *Sneathia sanguinegens* (CCUG 42621), *Sneathia amnii* (CCUG 52977 T), *Lactobacillus gasseri* (CCUG 44082), *Bifidobacterium breve* (CCUG 27171 A), and *Escherichia coli* (CCUG 44113). Except *Escherichia coli* grow with LB medium under aerobic condition, all bacterial strains were cultured anaerobically using NYCIII medium.

For the co-culture experiment, 2 × 10⁵ HeLa cells or VK2 cells were plated in a 12-well plate and incubated overnight at 37 °C with 5% CO₂. Bacterial culture after 24 to 48 hours were diluted in DMEM and added to the HeLa cells at a multiplicity of infection (MOI) of 10, after adjusting the bacterial optical density (OD). After 6 and 24 hours of co-culturing, supernatants were collected and used for subsequent assays.

### Enzyme-Linked Immunosorbent Assay (ELISA) Assay

Cytokine concentrations in the supernatants were measured using ELISA kits, with reagents and antibodies purchased from PeproTech® and Fisher Scientific (USA). Each sample was tested in triplicate and repeated at least two times. Cytokines included IL-8, IL-6, IL-1 alpha, and IL-1 beta, and their information of capture antibodies and detection antibodies showed in Supplementary Table 2. The ELISA plates were measured using the SpectraMax MiniMax™ 300 Imaging Cytometer (Molecular Devices, USA) with a 450 nm.

### Statistical analysis

The Kruskal-Wallis test was employed to compare microbial alpha and beta diversities more than two groups, and Benjamini-Hochberg correction was applied to adjust for multiple testing and reduce the false discovery rate. Pearson analysis was used to analyze heatmap and network data. Cytokine concentrations from ELISA tests were analyzed by One-way ANOVA and T-test. The relationship between HPV infection, vaginal microbiota, and age was assessed using the χ² (Chi-square) test. Alpha diversity was calculated with QIIME (Version 1.7.0) and displayed with R software (Version 2.15.3). Beta diversity on unweighted unifrac were calculated by QIIME software (Version 1.9.1). PCoA analysis was displayed by the WGCNA package, stat packages and ggplot2 package in R software(Version 2.15.3). Random forest was displayed with R software (Version 3.2.1) randomForest package. Terneryplot was displayed with R software (Version 2.15.3) Rvcd package ternaryplot command. Heatmap and PCA were displayed with GraphPad Prism. Network was displayed with Flourosh website (https://app.flourish.studio/templates). All statistical analyses were conducted using SPSS version 27 (IBM, New York, NY). A *p*-value of less than 0.05 was considered statistically significant. Graphical images were generated using GraphPad Prism 9.0.

## Data availablity

The datasets presented in this study can be found in online repositories. The names of the repository/repositories and accession number can be found below: NCBI; PRJNA787754.

## Supplementary information

Supplementary Information file

## Data Availability

The datasets presented in this study can be found in online repositories. The names of the repository/repositories and accession number can be found below: NCBI; PRJNA787754.
