# Supplementary material for: Vaginal microbiome and inflammation cytokines among Chinese women
Source: NPJ Biofilms Microbiomes. 2026 May 20;12:151. doi: 10.1038/s41522-026-00994-w (PMC13454521; doi:10.1038/s41522-026-00994-w)
Supplement: Supplementary file 1 [file 41522_2026_994_MOESM1_ESM.pdf]

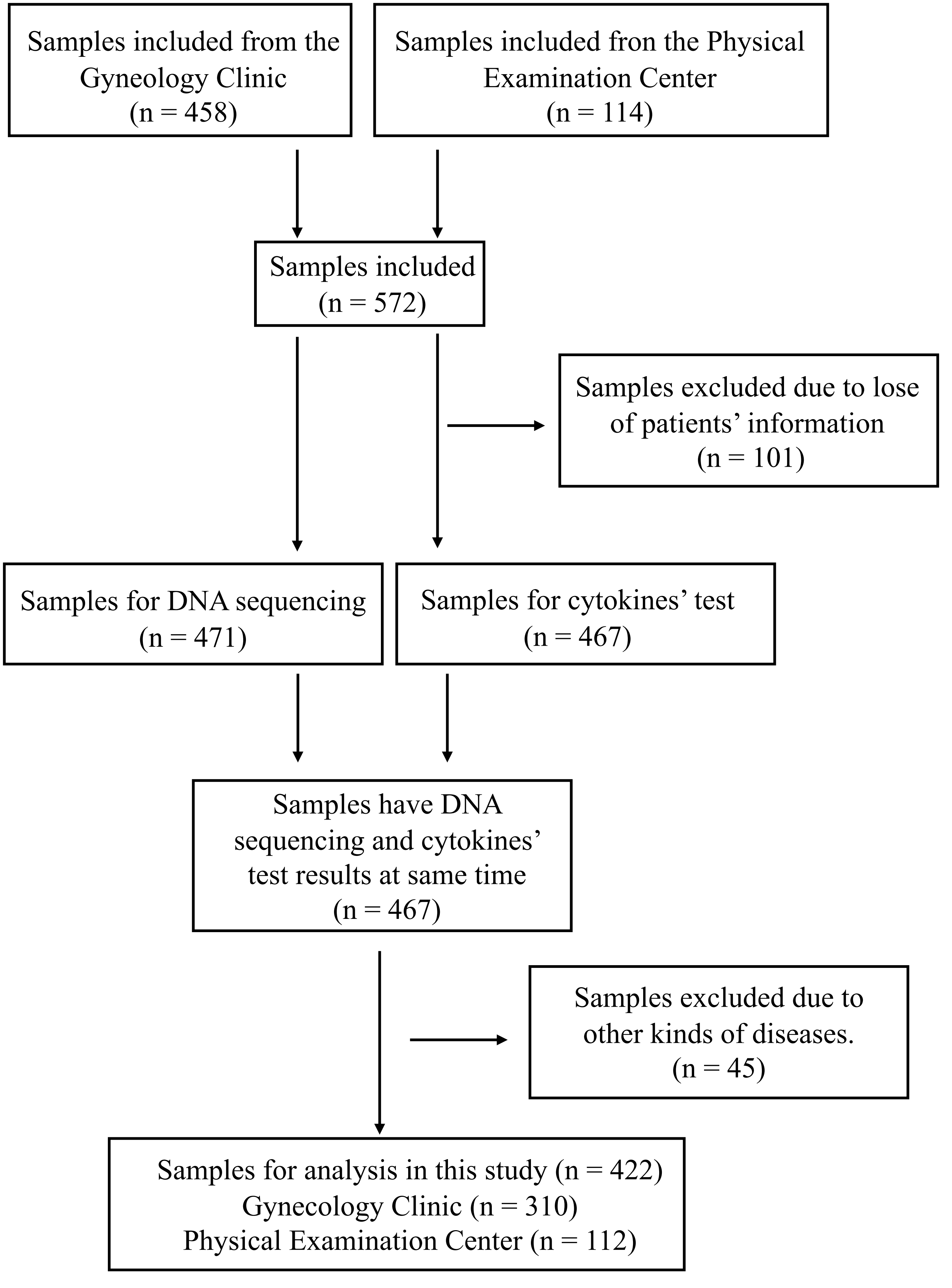

Supplementary Figure 1: Overview of clinical samples

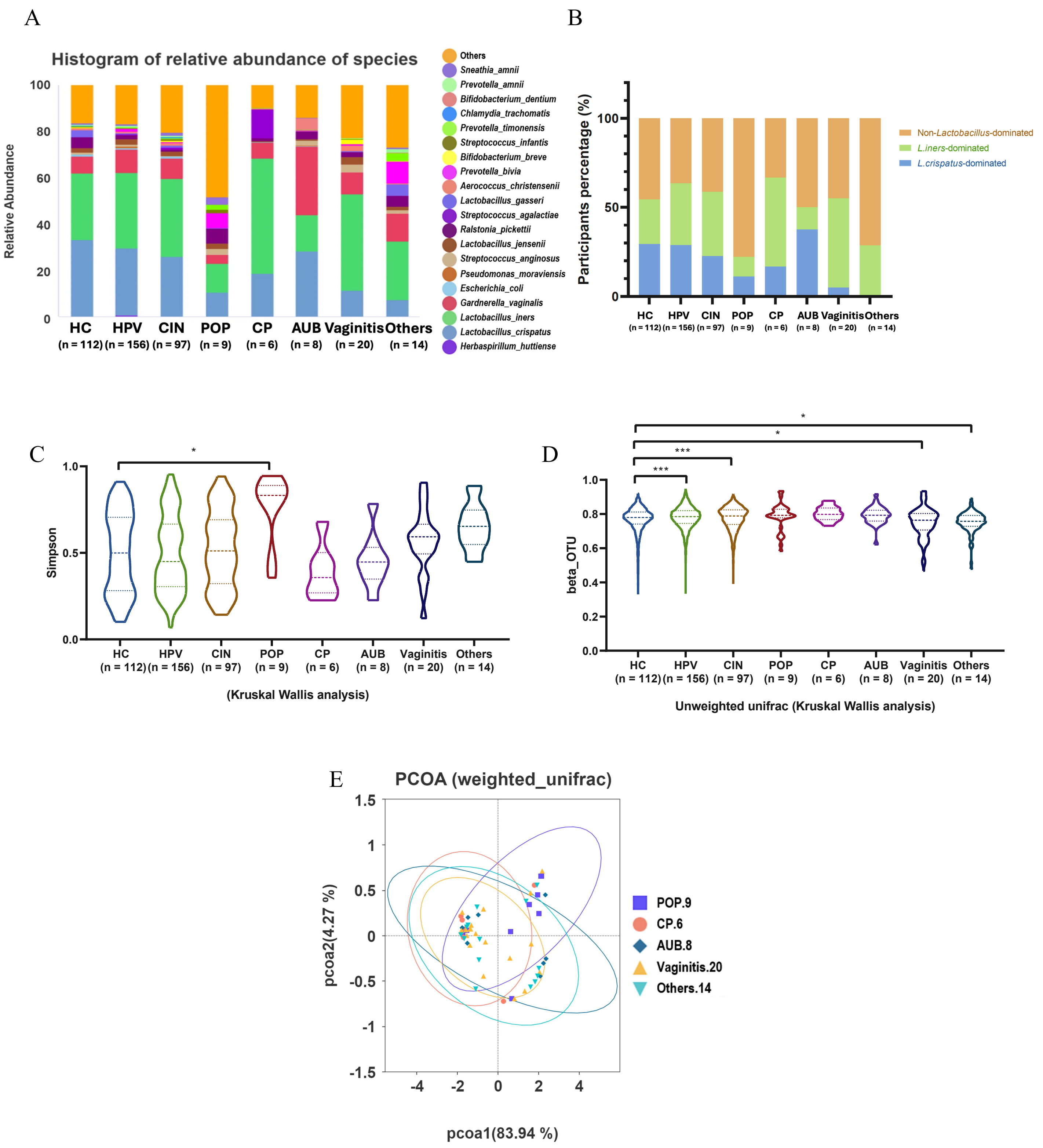

Supplementray Figure 2: Vaginal microbiota (VMB) of all the participants . A) Histogram of relative abundance of eight conditions including healthy individuals (HC), Human papillomavirus (HPV), cervical intraepithelial neoplasia (CIN), pelvic organ prolapse (POP), cervical polyps (CP), abnormal uterine bleeding (AUB), vaginitis, and other diseases. **B)** Bar chart of participants percentage in eight groups, which was divided by *L.crispatus* – dominated, *L. iners* – dominated, and Non – *Lactobacillus* – dominated with 50% as the dominate cut off. (Pearson k^2 test, $p = 0.039$, Pearson $k^2 = 24.594$). **C)** Simpson alpha diversity analysis of all the eight conditions ($p_{(HC-POP)}$: 0.031). **D)** Beta diversity analysis of the OUT in eight groups with unweighted unifrac distance algorithm presented by violin blot ($p_{(HC-HPV)}$: < 0.0001, $p_{(HC-CIN)}$: <0.0001, $p_{(HC-Vaginitis)}$: 0.034, $p_{(HC-Others)}$: 0.029). **E)** Beta diversity analysis of the OUT in eight groups with unweighted unifrac distance algorithm presented by PCoA (Principal Coordinates Analisis) (Figure 2C and 2D used Kruskal Wallis analysis, and Benjamini-Hochberg correction, p value has been adjusted, * : $p < 0.05$; ** : $p < 0.01$; *** : $p < 0.001$)

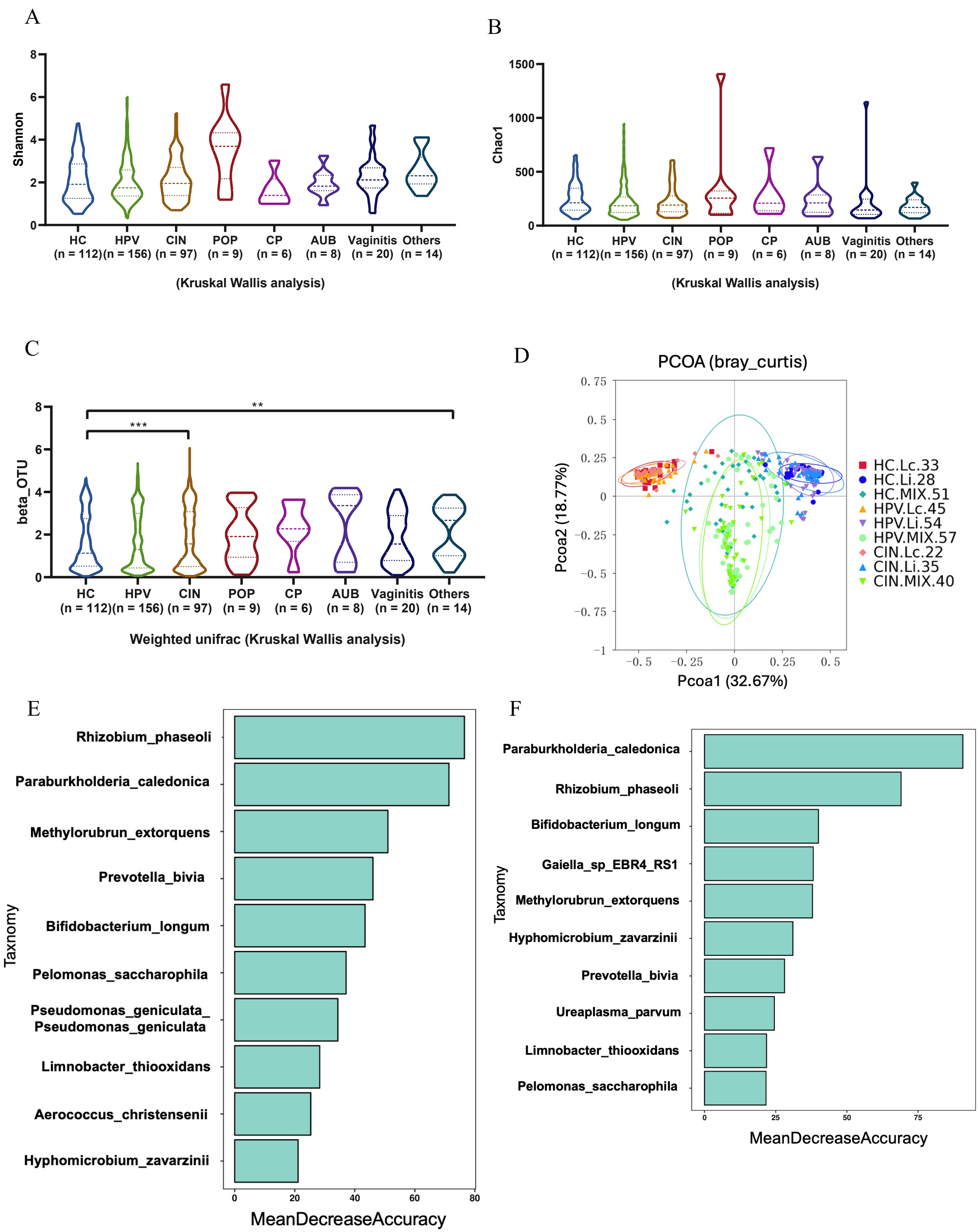

Supplementray Figure 3: Basic information of 422 clinical samples. A-B) Alpha analysis of all the clinical samples. **C)** Beta analysis of all the samples ($p_{(HC-CIN)}$: <0.0001, $p_{(HC-Others)}$: 0.002). **D)** PCoA result of all the samples. **E)** Random forest analysis identifying the top10 species by Mean Decrease in the HC and HPV groups. **F)** Random forest analysis identifying the top10 species by Mean Decrease in the HC and CIN groups. (Figure 4A, 4B, and 4C used Kruskal Wallis analysis, and Benjamini-Hochberg correction, p value has been adjusted, * : p < 0.05; ** : p < 0.01; *** : p < 0.001)

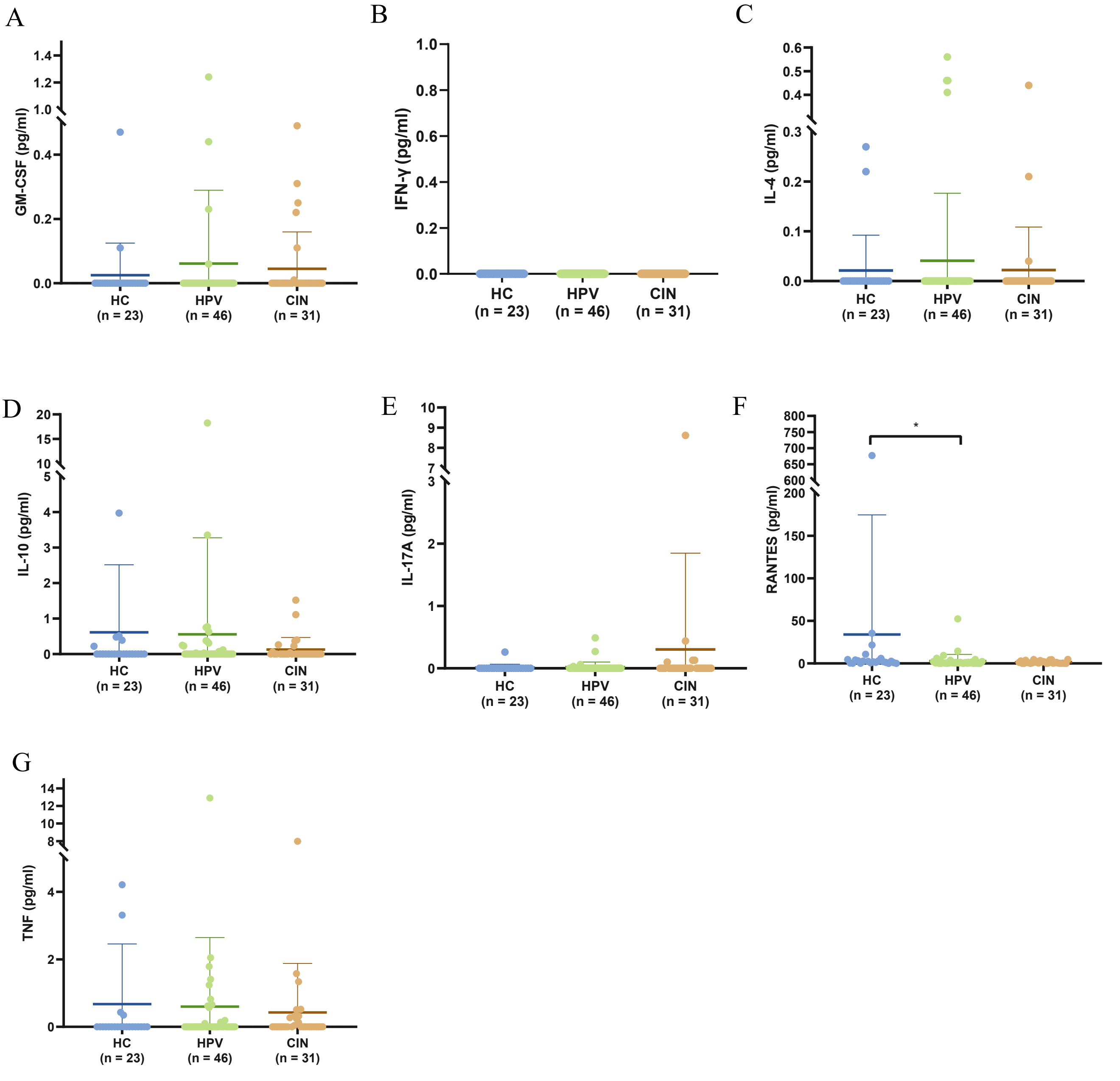

Supplementray Figure 4: Production of Cytokines in HC, HPV, and CIN groups. A-G) Concentrations of **A)** GM-CSF, **B)** IFN-γ, **C)** IL-4, **D)** IL-10, **E)** IL-17A, **F)** RANTES ($p_{(HC\text{-}HPV)}$: 0.046), and **G)** TNF were analyzed. (Figure 3A - 3G used Kruskal Wallis analysis, and Benjamini-Hochberg correction, p value has been adjusted, * : p < 0.05; ** : p < 0.01; *** : p < 0.001)

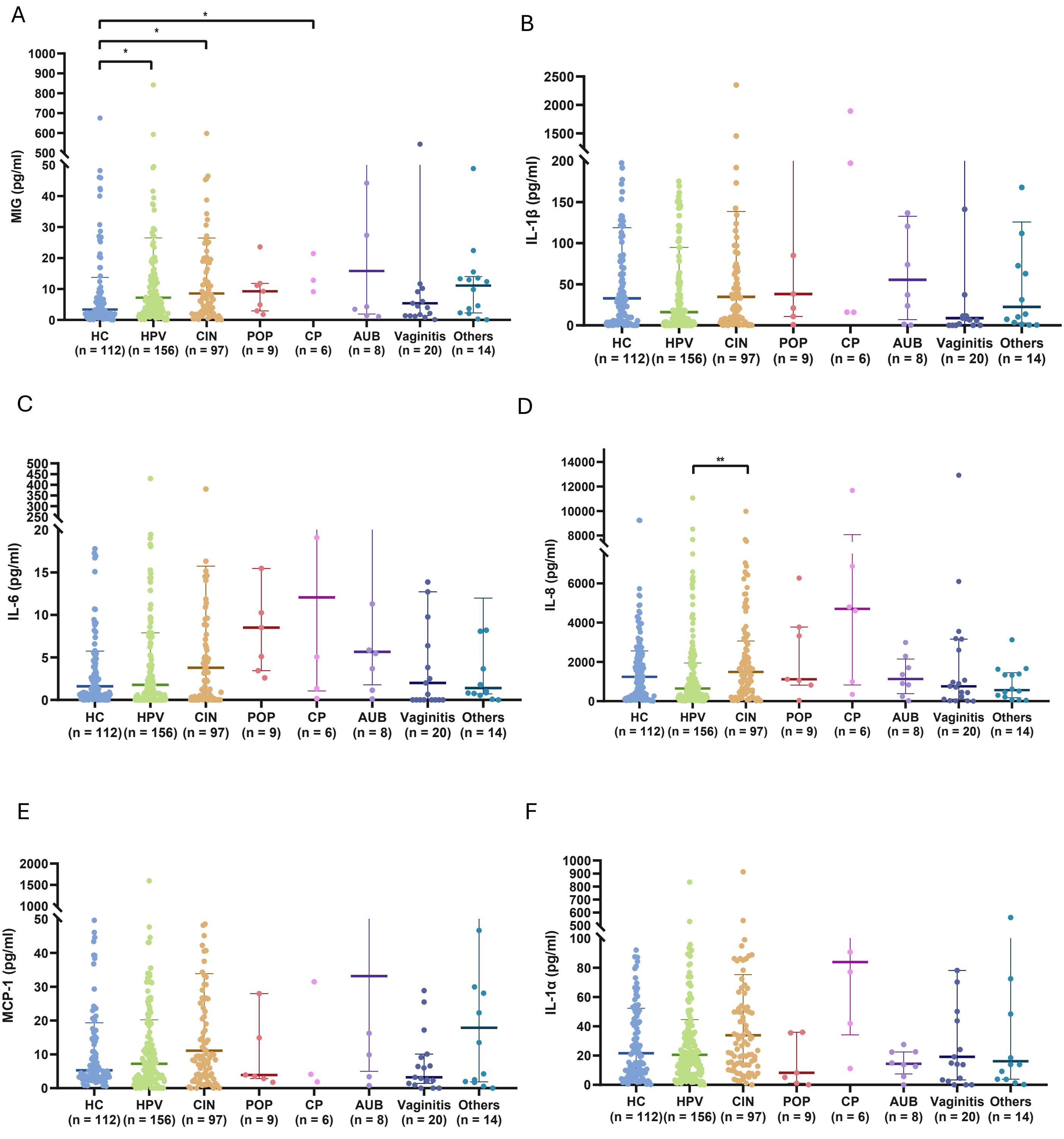

Supplementray Figure 5: Cytokine expression in HC, HPV, CIN, POP, CP, AUB, Vaginitis, and Others groups. A-F) Concentrations of **A)** MIG ($p_{(HC\text{-}HPV)}$: 0.027, $p_{(HC\text{-}CIN)}$: 0.035, $p_{(HC\text{-}CP)}$: 0.039), **B)** IL-1β, **C)** IL-6, **D)** IL-8 ($p_{(HPV\text{-}CIN)}$: 0.04), **E)** MCP-1, and **F)** IL-1α. (Figure 5A - 5F used Kruskal Wallis analysis, and Benjamini-Hochberg correction, p value has been adjusted, * : $p < 0.05$; ** : $p < 0.01$; *** : $p < 0.001$)

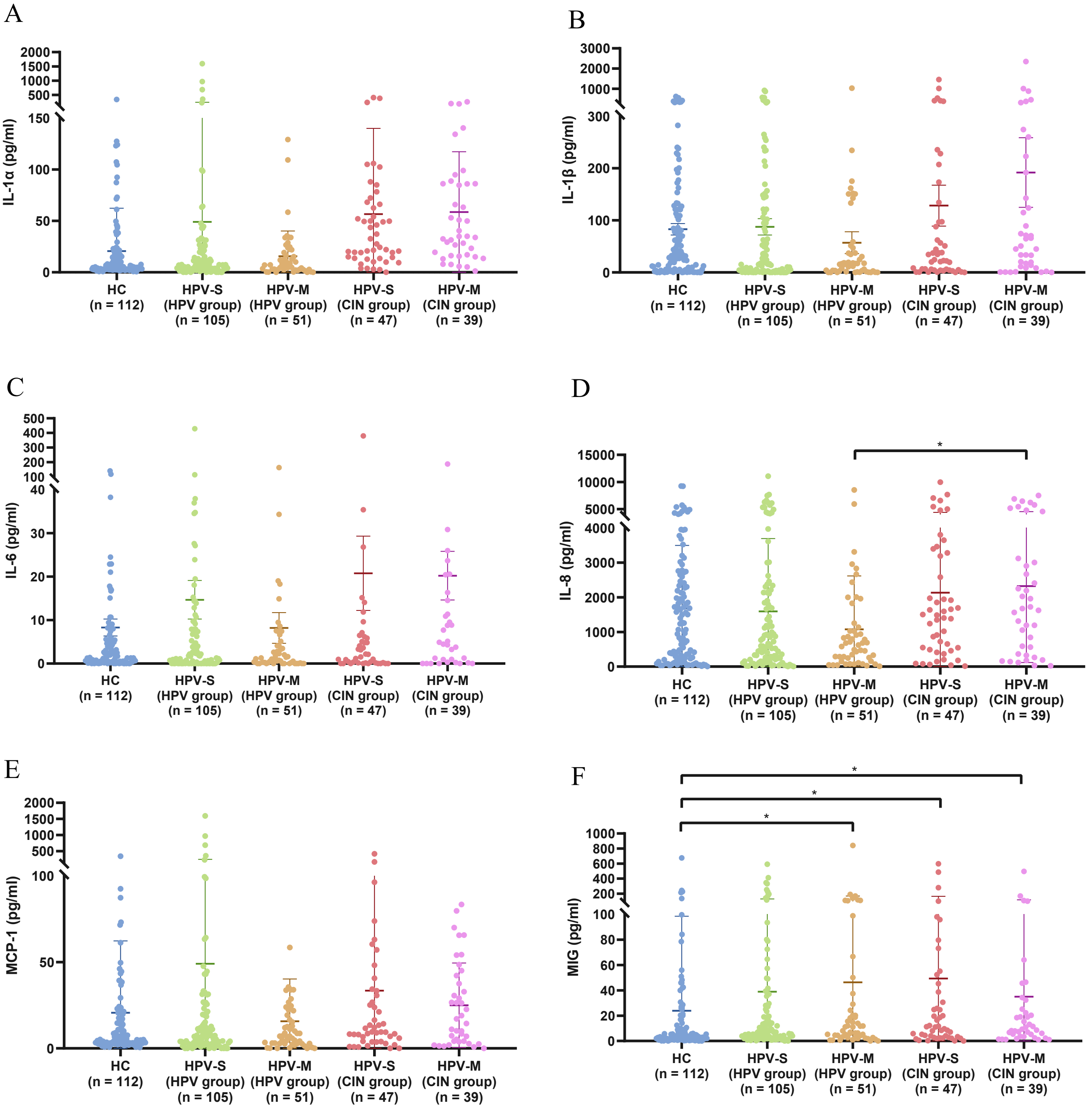

Supplementary Figure 6: Cytokines expression in healthy group, HPV single infection group, HPV multiple infection group, HPV single infection in CIN group, and HPV multiple infection in CIN group. A-F) Level of **A)** IL-1alpha, **B)** IL-1beta, **C)** IL-6, **D)** IL-8 ($p_{\text{(HPV-M (HPV group) – HPV-M (CIN group))}}$: 0.027), **E)** MCP-1, and **F)** MIG ($p_{\text{(HC - HPV-M(HPV group))}}$: 0.044, $p_{\text{(HC - HPV-S (CIN group))}}$: 0.018, $p_{\text{(HC – HPV-M (CIN group))}}$: 0.029). (Figure 10A – 10F used Kruskal Wallis analysis, and Benjamini-Hochberg correction, p value has been adjusted, * : p < 0.05; ** : p < 0.01; *** : p < 0.001)

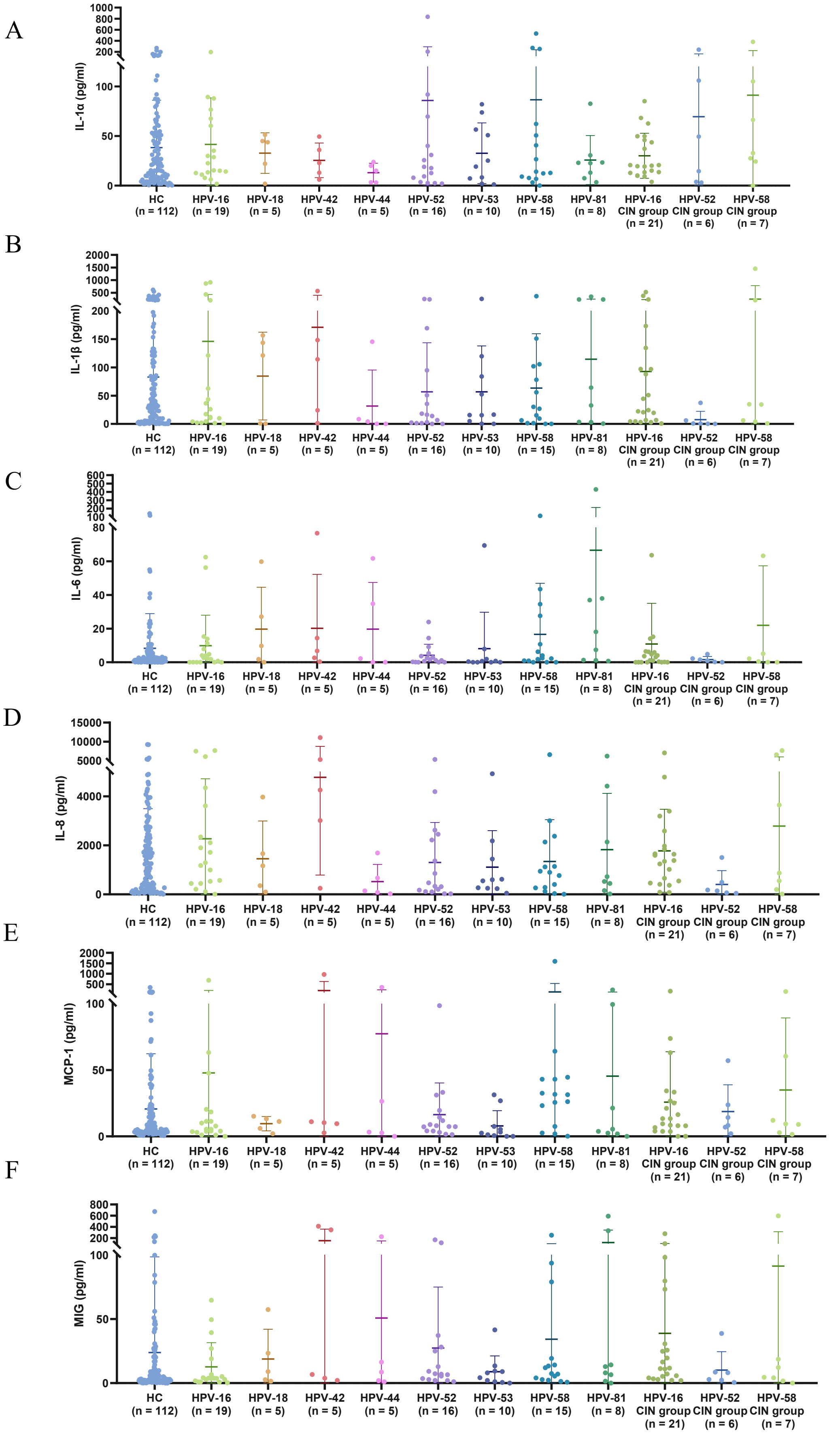

Supplementary Figure 7: Cytokines expression in HPV single genotype infection group. A-F) Level of **A)** IL-1alpha, **B)** IL-1beta, **C)** IL-6, **D)** IL-8, **E)** MCP-1, and **F)** MIG. (Figure 11A -11 F used Kruskal Wallis analysis, and Benjamini-Hochberg correction, p value has been adjusted, * : $p < 0.05$; ** : $p < 0.01$; *** : $p < 0.001$)

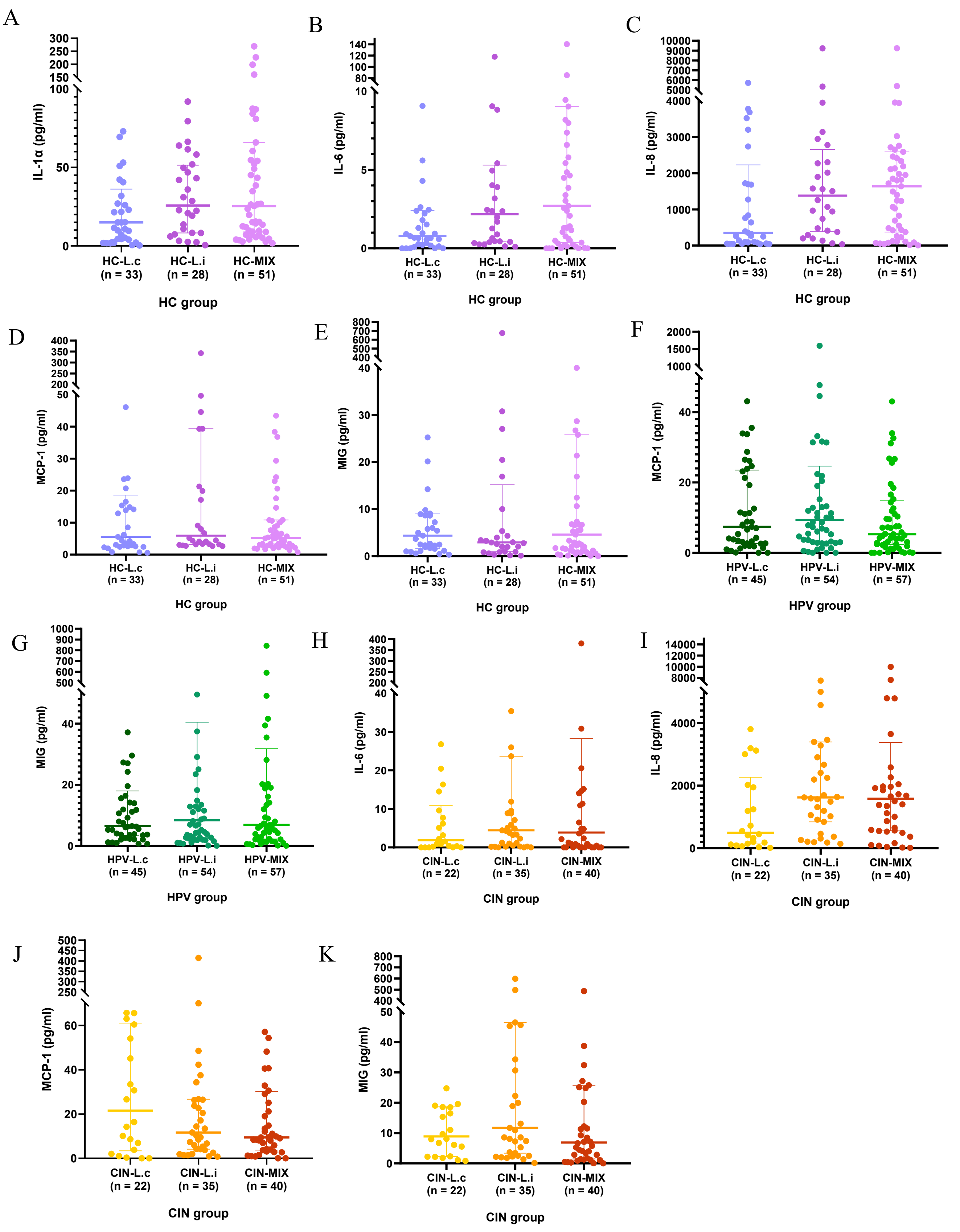

Supplementary Figure 8: Cytokine concentration in HC, HPV and CIN groups. A-E) Level of **A)** IL-1a, **B)** IL-6, **C)** IL-8**, D)** MCP-1, and **E)** MIG in the HC group. **F)** Level of **F)** MCP-1and **G)** MIG in the HPV group. **H-K)** Level of **H)** IL-6, **I)** IL-8, **J)** MCP-1, and **K)** MIG in the CIN group. (Figure 6A - 6K used Kruskal Wallis analysis, and Benjamini-Hochberg correction, p value has been adjusted, * : $p < 0.05$; ** : $p < 0.01$; *** : $p < 0.001$)

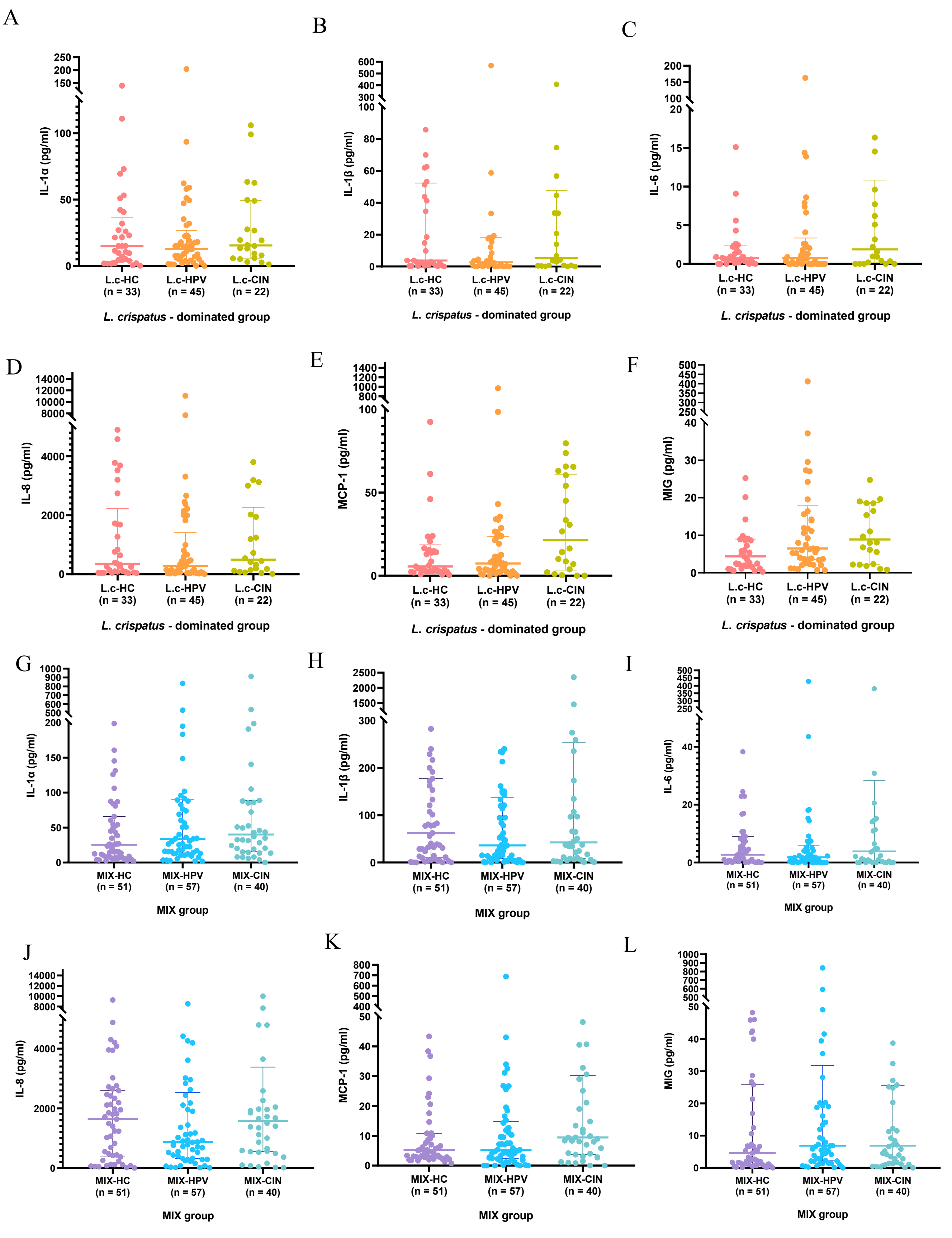

Supplementary Figure 9: Cytokine concentration in *L.crispatus*-dominated and MIX groups. A-F) No significant differences were observed in the concentrations of the tested six cytokines in *L.crispatus*-dominated group. **G-L)** No significant differences were observed in the concentrations of the tested six cytokines in Mix group. (Figure 7A – 7L used Kruskal Wallis analysis, and Benjamini-Hochberg correction, p value has been adjusted, * : $p < 0.05$; ** : $p < 0.01$; *** : $p < 0.001$)

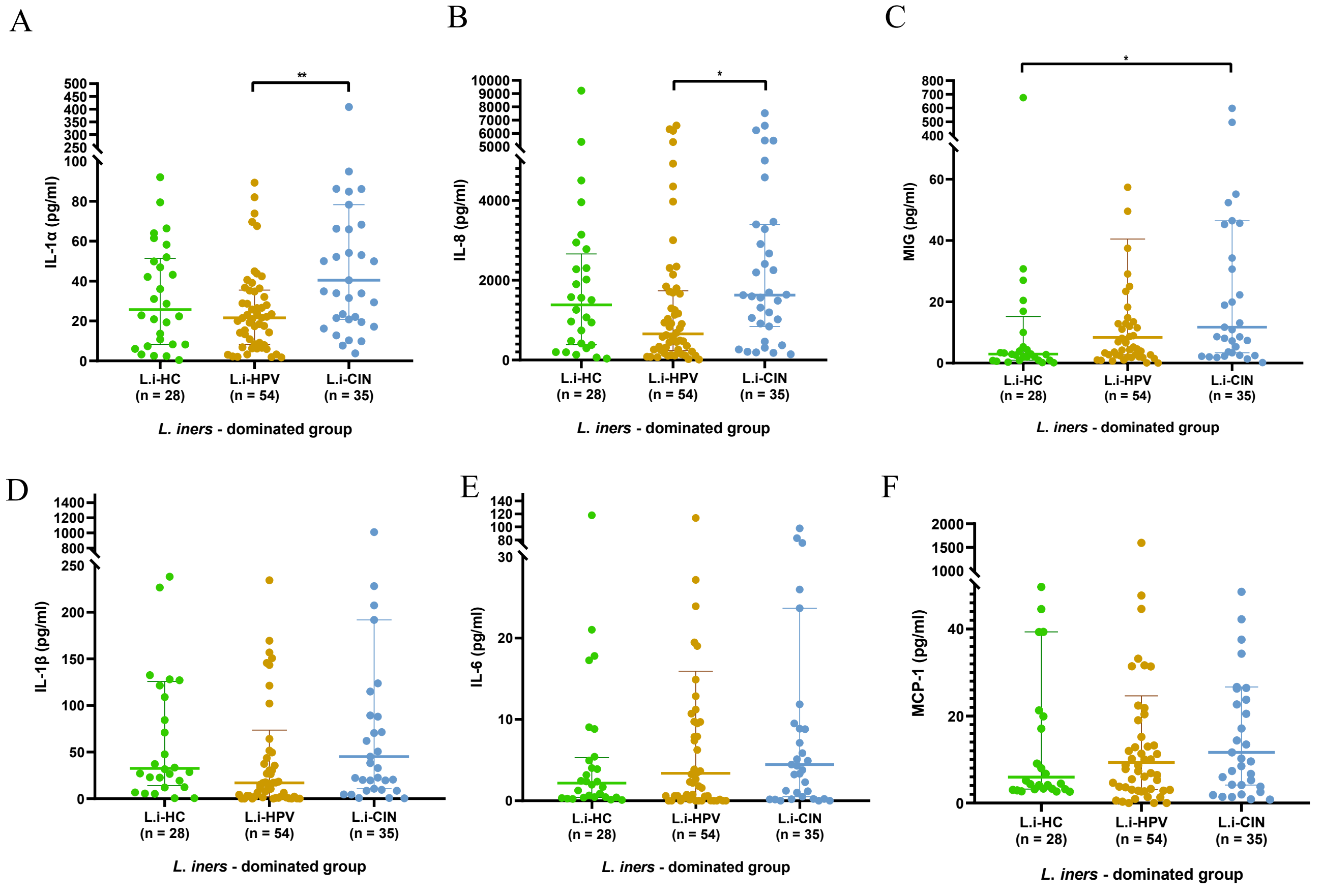

Supplementary Figure 10: Cytokine concentrations in *L.iners*-dominated group. A-F) Level of **A)** IL-1a (p $_{(HPV-CIN)}$: 0.003), **B)** IL-8 (p $_{(HPV-CIN)}$: 0.015), **C)** MIG (p $_{(HC-CIN)}$: 0.021), **D)** IL-1b, **E)** IL-6, and **F)** MCP-1 in the *L.iners*-dominated group. (Figure 8A – 8F used Kruskal Wallis analysis, and Benjamini-Hochberg correction, p value has been adjusted, * : $p < 0.05$; ** : $p < 0.01$; *** : $p < 0.001$)

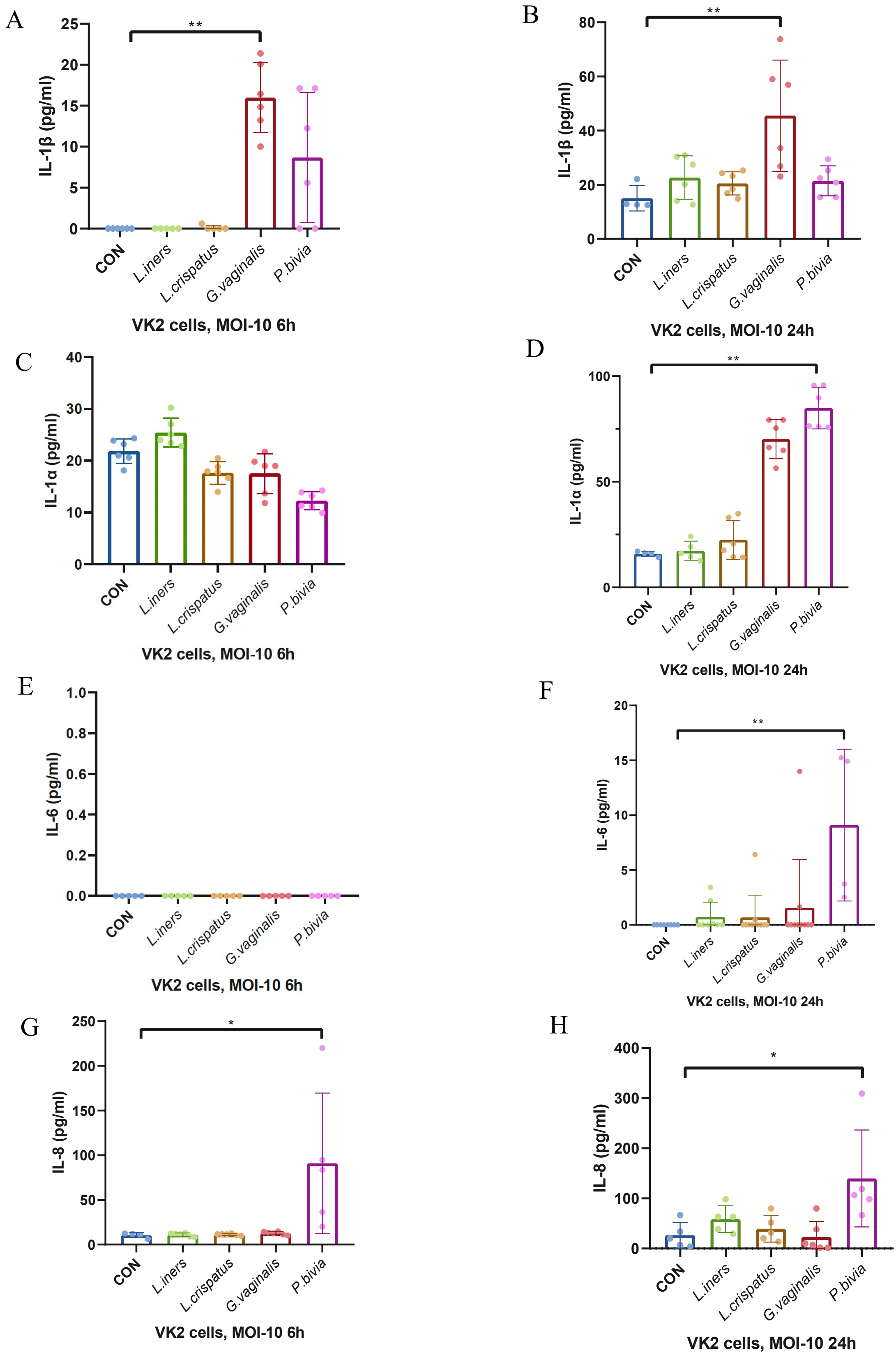

Supplementary Figure 11: Cytokines concentration tested by ELISA after co-culture with VK2 cells 6 and 24 hours. A) IL-1β concentration of 6 hours co-cultivation ($p_{(Control – G.vaginalis)}$: 0.003). **B)** IL-1β concentration of 24 hours co-cultivation ($p_{(Control – G.vaginalis)}$: 0.005). **C)** IL-1α concentration of 6 hours co-cultivation. **D)** IL-1α concentration of 24 hours co-cultivation ($p_{(Control – P.bivia)}$: 0.008). **E)** IL-6 concentration of 6 hours co-cultivation. **F)** IL-6 concentration of 24 hours co-cultivation ($p_{(Control – P.bivia)}$: 0.001). **G)** IL-8 concentration of 6 hours co-cultivation ($p_{(Control – P.bivia)}$: 0.036). **H)** IL-8 concentration of 24 hours co-cultivation ($p_{(Control – P.bivia)}$: 0.038). (Figure 9A – 9H used Kruskal Wallis analysis, and Benjamini-Hochberg correction, p value has been adjusted * : $p < 0.05$; ** : $p < 0.01$; *** : $p < 0.001$)

Supplementary Table 1: Information of antibodies in Cytometric Bead Array

Antibodies' name	Cat.No	Bead's Position	Sensitivity	Testing Unit
Hu GM-CSF CBA Flex Set C9 100Tst	558335	C9	0.2 pg/mL	10-2,500 pg/mL
Hu IFN-Gma CBA Flex Set B8 100Tst	560111	B8	0.8 pg/ml	10-2,500 pg/mL
Hu IL-1 Alp CBA Flex Set D6 100Tst	560153	D6	1.0 pg/mL	10-2,500 pg/mL
Hu IL-1Bta CBA Flex Set B4 100Tst	558279	B4	2.3 pg/mL	10-2,500 pg/mL
Hu IL-4 CBA Flex Set A5 100Tst	558272	A5	1.4 pg/mL	10-2,500 pg/mL
Hu IL-6 CBA Flex Set A7 100Tst	558276	A7	1.6 pg/mL	10-2,500 pg/mL
Hu IL-8 CBA Flex Set A9 100Tst	558277	A9	1.2 pg/mL	10-2,500 pg/mL
Hu IL-10 CBA Flex Set B7 100Tst	558274	B7	0.13 pg/mL	10-2,500 pg/mL
Hu IL-17A CBA Flex Set B5 100Tst	560383	B5	0.3 pg/mL	10-2,500 pg/mL
Hu MCP-1 CBA Flex Set D8 100Tst	558287	D8	1.3 pg/mL	10-2,500 pg/mL
Hu MIG CBA Flex Set E8 100Tst	558286	E8	1.1 pg/mL	10-2,500 pg/mL
Hu RANTES CBA Flex Set D4 100Tst	558324	D4	0.002 pg/mL	10-2,500 pg/mL
Hu TNF CBA Flex Set C4 100Tst	560112	C4	1.2 pg/ml	10-2,500 pg/mL

Supplementary Table 2: Information of antibodies in Enzyme-Linked Immunosorbent Assay

	Capture Antibody	Detection Antibody
**IL-6**	Monoclonal Antibody<br>(Cat.No: 500-M06-500UG)	Polyclonal Antibody, Biotin<br>(Cat. No: 500-P26GBT-50UG)
**IL-8**	Monoclonal Antibody<br>(Cat.No: 500-M08-500UG)	Polyclonal Antibody, Biotin<br>(Cat. No: 500-P28BT-50UG)
**IL-1 beta**	Monoclonal Antibody<br>(Cat.No: 500-M01B-500UG)	Polyclonal Antibody, Biotin<br>(Cat. No: 500-P21BGBT-50UG)
**IL-1 alpha**	Monoclonal Antibody (ILA9-H18)<br>(Cat.No: M421A)	Monoclonal Antibody (ILA8-H12) Biotin<br>(Cat. No: M420AB)
